# Supplementary material for: PAX3-FOXO1 dictates myogenic reprogramming and rhabdomyosarcoma identity in endothelial progenitors
Source: Nat Commun. 2023 Nov 15;14:7291. doi: 10.1038/s41467-023-43044-1 (PMC10651858; doi:10.1038/s41467-023-43044-1)
Supplement: Supplementary file 1 — Supplementary Information [file 41467_2023_43044_MOESM1_ESM.pdf]

SUPPLEMENTARY INFORMATION

PAX3-FOXO1 dictates myogenic reprogramming and rhabdomyosarcoma identity in endothelial progenitors

Madeline B. Searcy[1,2,†], Randolph K. Larsen IV[1,2,†], Bradley T. Stevens[1,2], Yang Zhang[3], Hongjian Jin[4], Catherine J. Drummond[1], Casey G. Langdon[1], Katherine E. Gadek[1], Kyna Vuong[1], Kristin B. Reed[1], Matthew R. Garcia[1], Beisi Xu[4], Darden W. Kimbrough[1,5], Grace E. Adkins[1,2], Nadhir Djekidel[4], Shaina N. Porter[6], Patrick A. Schreiner[3], Shondra M Pruett-Miller[6], Brian J. Abraham[3], Jerold E. Rehg[7], Mark E. Hatley[1]*

[1] Department of Oncology, St. Jude Children's Research Hospital, Memphis, TN, 38105, USA

[2] St. Jude Graduate School of Biomedical Sciences, Memphis, TN, 38105, USA

[3] Department of Computational Biology, St. Jude Children's Research Hospital, Memphis, TN, 38105, USA

[4] Center for Applied Bioinformatics, St. Jude Children's Research Hospital, Memphis, TN, 38105, USA

[5] Rhodes College, Memphis, TN, 38112, USA

[6] Department of Cell and Molecular Biology, St. Jude Children's Research Hospital, Memphis, TN, 38105, USA

[7] Department of Pathology, St. Jude Children's Research Hospital, Memphis, TN, 38105, USA

† These authors contributed equally to this work

* Address correspondence to: Mark E. Hatley, M.D., Ph.D. (mark.hatley@stjude.org)

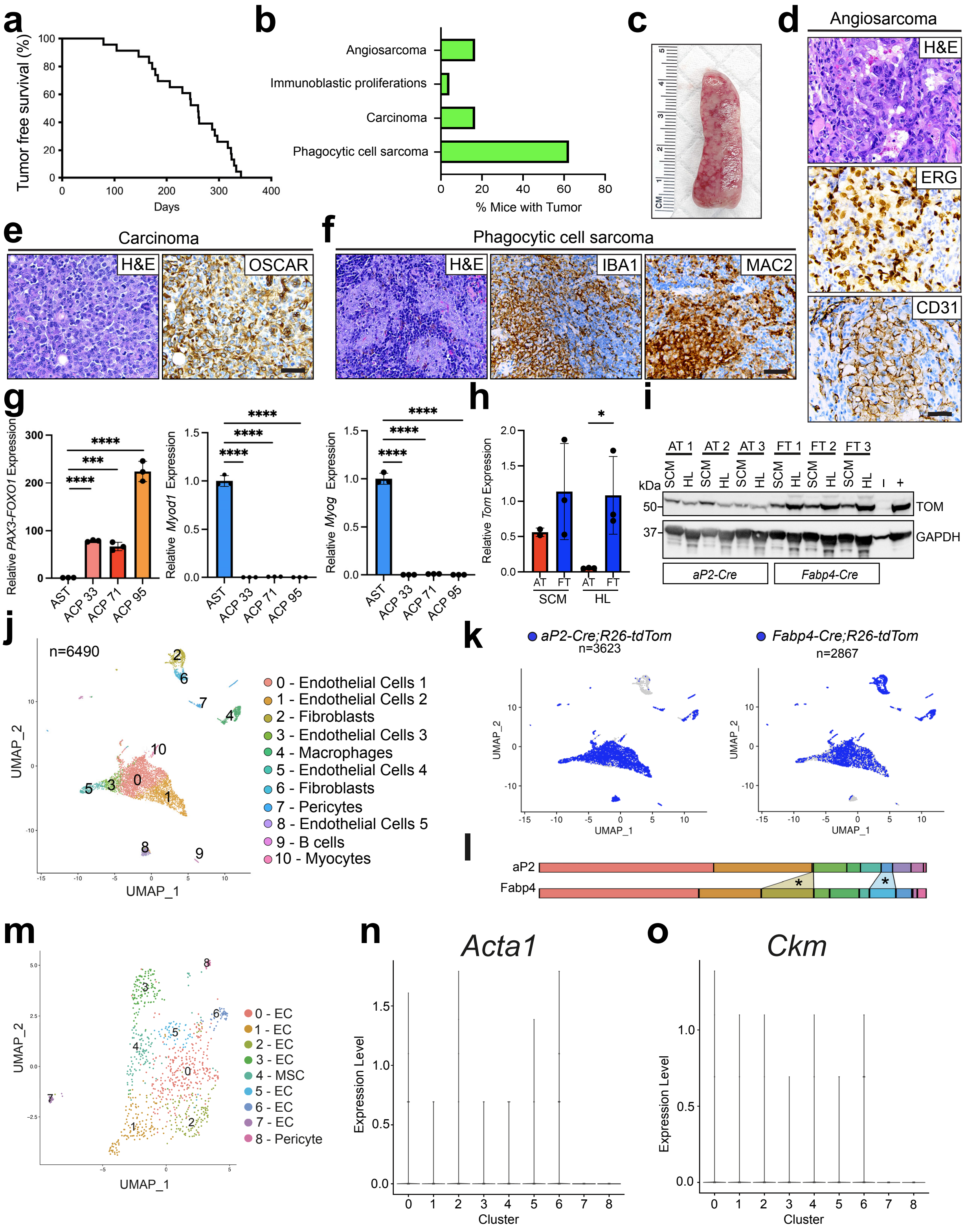

Supplementary Figure S1 Searcy and Larsen et al.

p

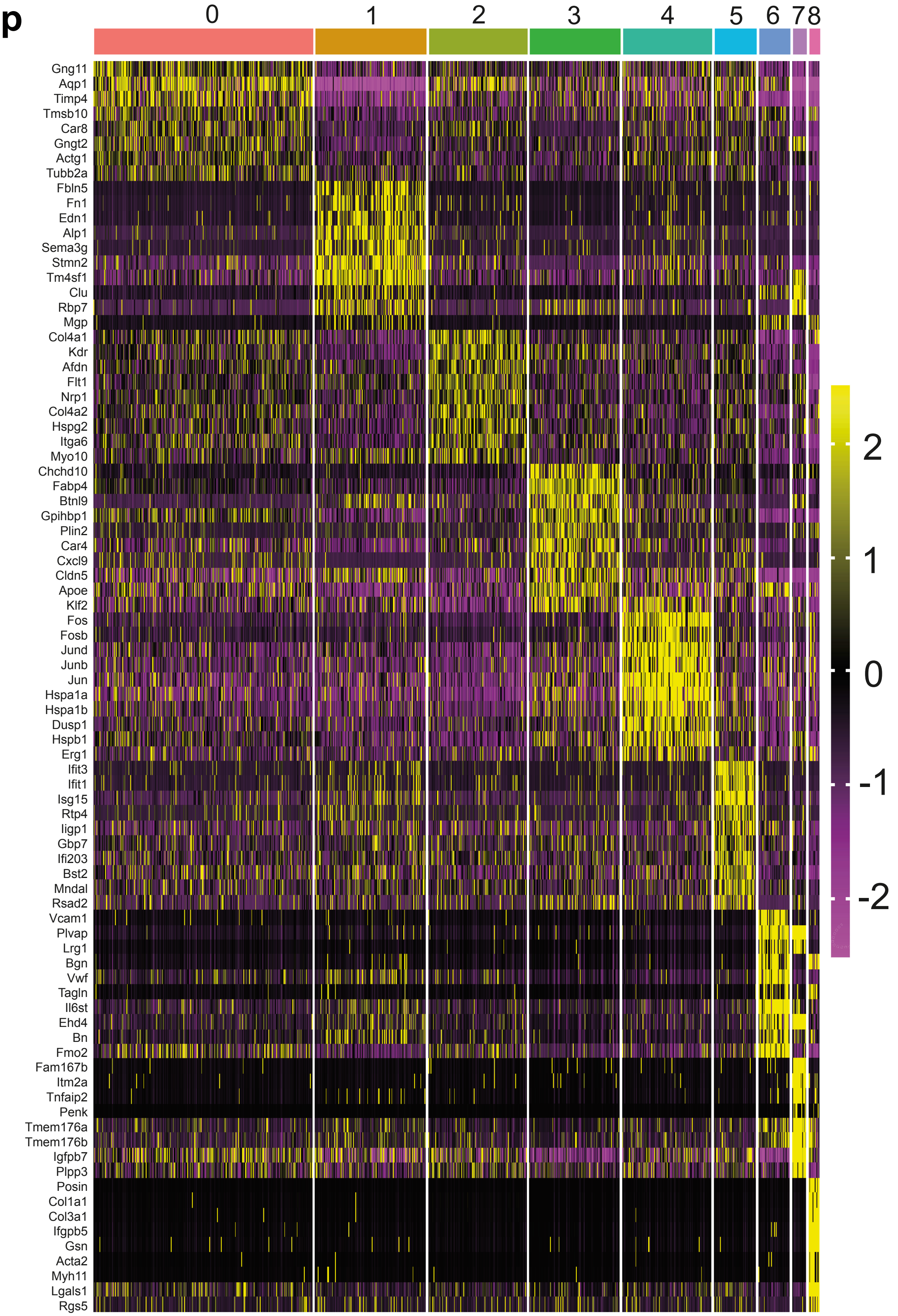

Supplementary Figure S1_2 Searcy and Larsen et al.

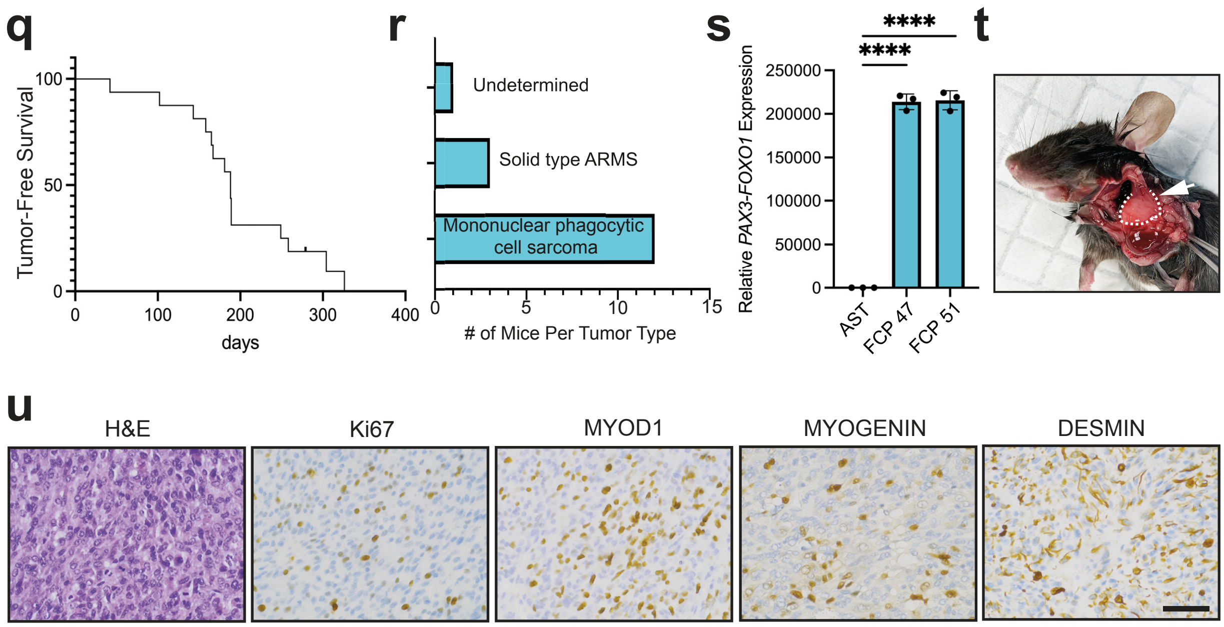

Supplementary Figure 1. P3F expression and *Cdkn2a* loss transforms *aP2-Cre* and *Fabp4-Cre* expressing endothelial cells. **a** Kaplan-Meier tumor-free survival in ACP mice (n = 25). **b** Tumor types arising in ACP mice. **c** Enlarged spleen with phagocytic cell sarcoma in ACP mice. **d** Representative histology of angiosarcoma tumors in ACP mice (n = 3). Scale bar = 40μm. **e** Representative histology of mononuclear phagocytic cell sarcoma tumors in ACP mice (n = 16). Scale bar = 40 μm. **f** Representative histology of carcinoma tumors in ACP mice (n = 3). Scale bar = 40 μm. **g** Gene expression by real-time PCR for *Pax3-Foxo1* ("ACP 33" P < 0.0001, "ACP 71" P = 0.0002, "ACP 95" P < 0.0001), *Myod1* ("ACP 33" P < 0.0001, "ACP 71" P < 0.0001, "ACP 95" P < 0.0001) and *Myogenin* ("ACP 33" P < 0.0001, "ACP 71" P < 0.0001, "ACP 95" P < 0.0001) in ACP tumors compared to FN-RMS mouse model *aP2-Cre;R26-SmoM2;R26-tdTomato* (AST)(3 ACP mice, 1 AST mouse, n = 3 technical replicates per tumor). **h** Gene expression by real-time PCR of *Tomato* in *aP2-Cre;R26-tdTom* (AT) and *Fabp4-Cre;R26-tdTom* (FT) SCM (n = 3 separate mice per genotype, P = 0.0315). **i** Immunoblots of TOMATO and GAPDH in *aP2-Cre;R26-tdTom* and *Fabp4-Cre;R26-tdTom* SCM. **j** UMAP and cluster analysis of single-cell transcriptional profiles of Tom^{+} cells from *aP2-Cre;R26-tdTom* (n = 2 experimental replicates) and *Fabp4-Cre;R26-tdTom* (n = 2 experimental replicates) SCM. **k** UMAP highlighted by sample. **l** Proportions of clusters present in *aP2-Cre;R26-tdTom* and *Fabp4-Cre;R26-tdTom* SCM. Significantly different (P < 0.05) clusters indicated *. **m** UMAP and cluster analysis of single-cell transcriptional profiles from the top 50% of Tom^{+} cells from *aP2-Cre;R26-tdTom* (n = 1 experimental replicate) SCM. **n** Violin plot of *Acta1* expression in each cluster. **o** Violin plot of *Ckm* expression in each cluster. **p** Heatmap of top expressed genes in each cluster. **q** Kaplan-Meier tumor-free survival in FCP mice (n = 16). **r** Tumor types in FCP mice. **s** *Pax3-Foxo1* expression in FCP mice compared to FN-RMS AST mouse model. **t** FP-RMS tumor outlined by white dotted line, connected to the SCM (white arrow). **u** Representative histology of ARMS in FCP mice (n = 3). Scale bar = 100 μm. All *P* values in pairwise comparisons were determined by Student's *t* test (unpaired, two-tailed); *P < 0.05, ***P < 0.001, ****P < 0.0001. Data represented as mean +/- SEM. Source data are provided as a Source Data file.

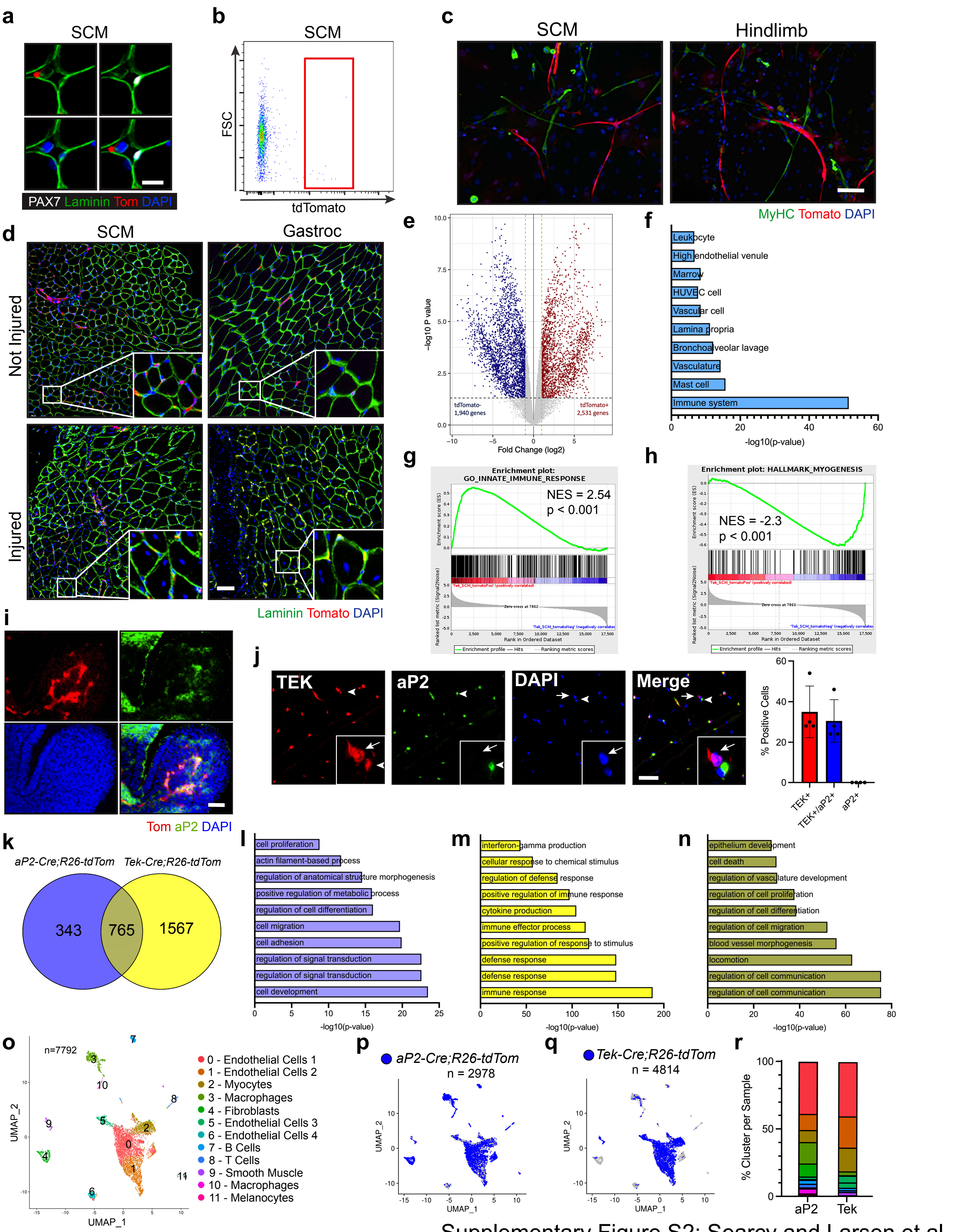

Supplementary Figure S2: Searcy and Larsen et al.

Supplementary Figure 2. *Tek-Cre* is not expressed by skeletal muscle cells. **a** Representative IF of *Tek-Cre;R26-tdTom* SCM skeletal muscle for PAX7 (white), Laminin (green), endogenous tdTomato fluorescence (red), and DAPI nuclear stain (blue) (n = 4 separate mice), Scale bar = 10 µm. **b** Representative flow cytometry of Tom^{+} cells from β1-Integrin+/CXCR4+ satellite cell population isolated from *Tek-Cre;R26-tdTom* SCM (n = 3 separate experimental replicates). **c** Representative IF of myogenic differentiation assay from *Tek-Cre;R26-tdTom* SCM and hindlimb skeletal muscle stained for MyHC (green), endogenous tdTomato (red), and DAPI nuclear stain (blue)(n = 3 experimental replicates), Scale bar = 100 µm. **d** Representative IF of CTX injured and not injured *Tek-Cre;R26-tdTom* SCM and gastroc skeletal muscle for Laminin (green), endogenous tdTomato fluorescence (red), and DAPI nuclear stain (blue)(n = 3 experimental replicates), Scale bar = 100 µm. **e** Volcano plot depicting differentially expressed genes P < (0.05) between Tom^{-} and Tom^{+} cells from *Tek-Cre;R26-tdTom* SCM skeletal muscle. **f** Top ten unique JENSEN tissue terms associated with upregulated genes from Tom^{+} cells from *Tek-Cre;R26-tdTom* SCM skeletal muscle. FDR < 0.05 and log2 fold-change > 1. **g** Enrichment plot of Innate Immune Response and **(h)** Enrichment plot of Hallmarks of Myogenesis on RNAseq results from Tom^{+} cells from *Tek-Cre;R26-tdTom* SCM skeletal muscle.. **i** Representative IF of branchial arches from embryonic day 10.5 (E10.5) *Tek-Cre;R26-tdTom* embryos stained for aP2 (green), endogenous Tomato (red), and DAPI nuclear stain (blue) Scale bar = 100 µm. **j** Representative IF of *Tek-Cre;R26-tdTom* SCM stained for aP2 (green), endogenous Tomato (red), and DAPI nuclear stain (blue) Scale bar = 50 µm. **k** Venn diagram showing genes with a two-fold increase in tomato positive cells compared to tomato negative cells from *aP2-Cre* (AT) and *Tek-Cre* (TT) SCM with P < 0.05. **l** Genes increased in AT. Top 10 unique GO terms shown. **m** Genes increased in TT. Top 10 unique GO terms shown. **n** Genes overlapping in AT and TT. Top 10 unique GO terms shown. **o** UMAP and cluster analysis of single-cell transcriptional profiles of tomato positive cells collected by FACS from *aP2-Cre* and *Tek-Cre* SCM skeletal muscle. **p, q** UMAPs highlighted by sample in blue from (O). **r** Proportions of populations present in each sample from (O). Source data are provided as a Source Data file.

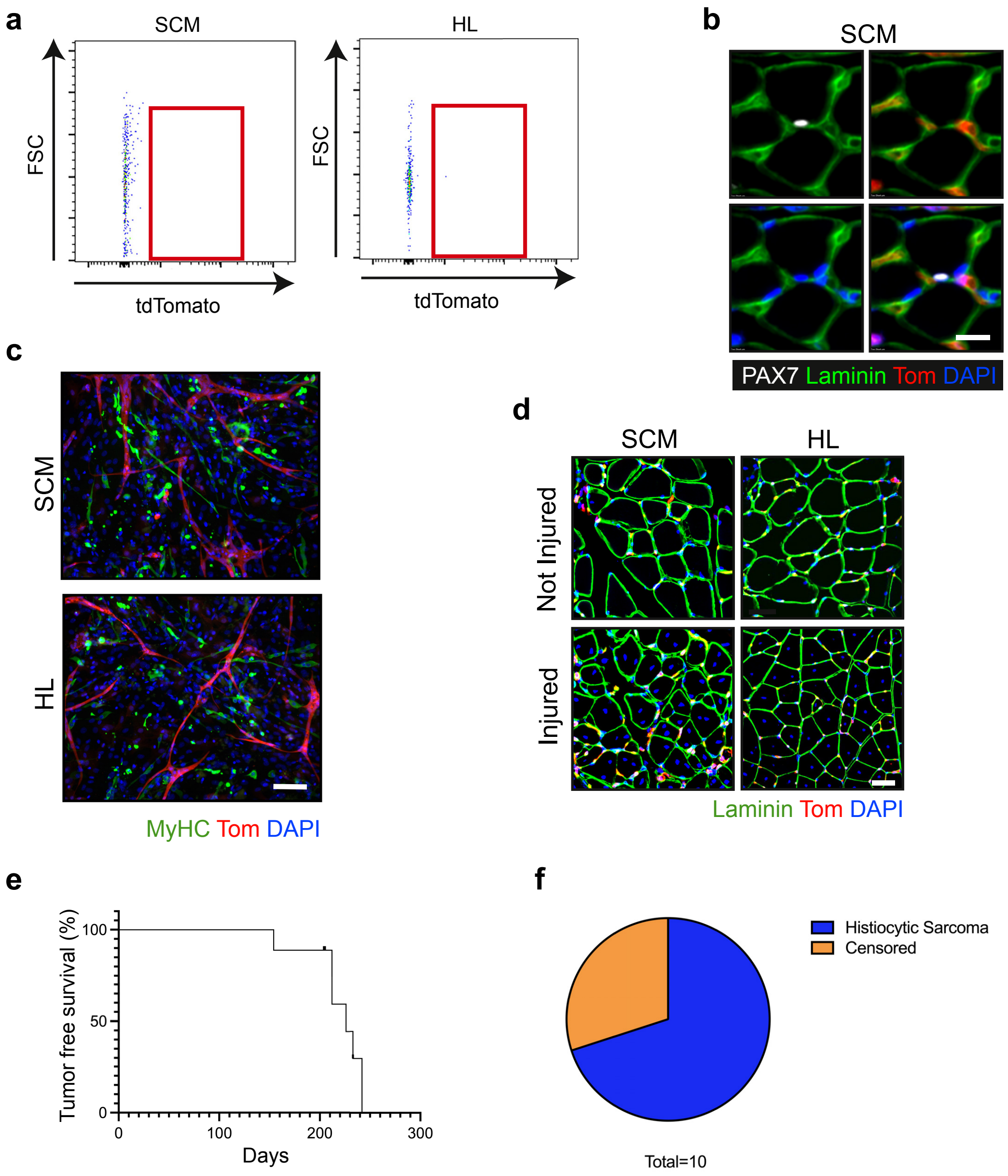

Supplementary Figure 3. *Tek-Cre;Cdkn2a*$^{Flox/Flox}$*;Pax*$^{P3Fm/P3Fm}$ cells are not muscle stem cells. **a** Representative flow cytometry of tomato positive cells from β1-Integrin+/CXCR4+ satellite cell population isolated from TCP SCM and hindlimb skeletal (n = 3 separate experimental replicates). **b** Representative IF of TCP SCM skeletal muscle for PAX7 (white), Laminin (green), endogenous tdTomato fluorescence (red), DAPI nuclear stain (blue)(n = 4 mice). Scale bar = 10 µm. **c** Representative IF of myogenic differentiation assay from cells isolated from TCP SCM and hindlimb skeletal muscle stained for MyHC (green), endogenous tdTomato fluorescence, DAPI nuclear stain (blue)(n = 3 separate replicates) Scale bar = 100µm. **d** Representative IF from CTX injured and uninjured TCP SCM and gastrocnemius skeletal muscle stained for Laminin (green), endogenous tdTomato fluorescence (red), DAPI nuclear stain (blue)(n = 3 mice). Scale bar = 25µm. **e** Kaplan-Meier tumor-free survival in *Tek-Cre;Cdkn2a*$^{flox/flox}$ mice (n = 10). **f** Tumor type of *Tek-Cre;Cdkn2a*$^{flox/flox}$ mice. Source data are provided as a Source Data file.

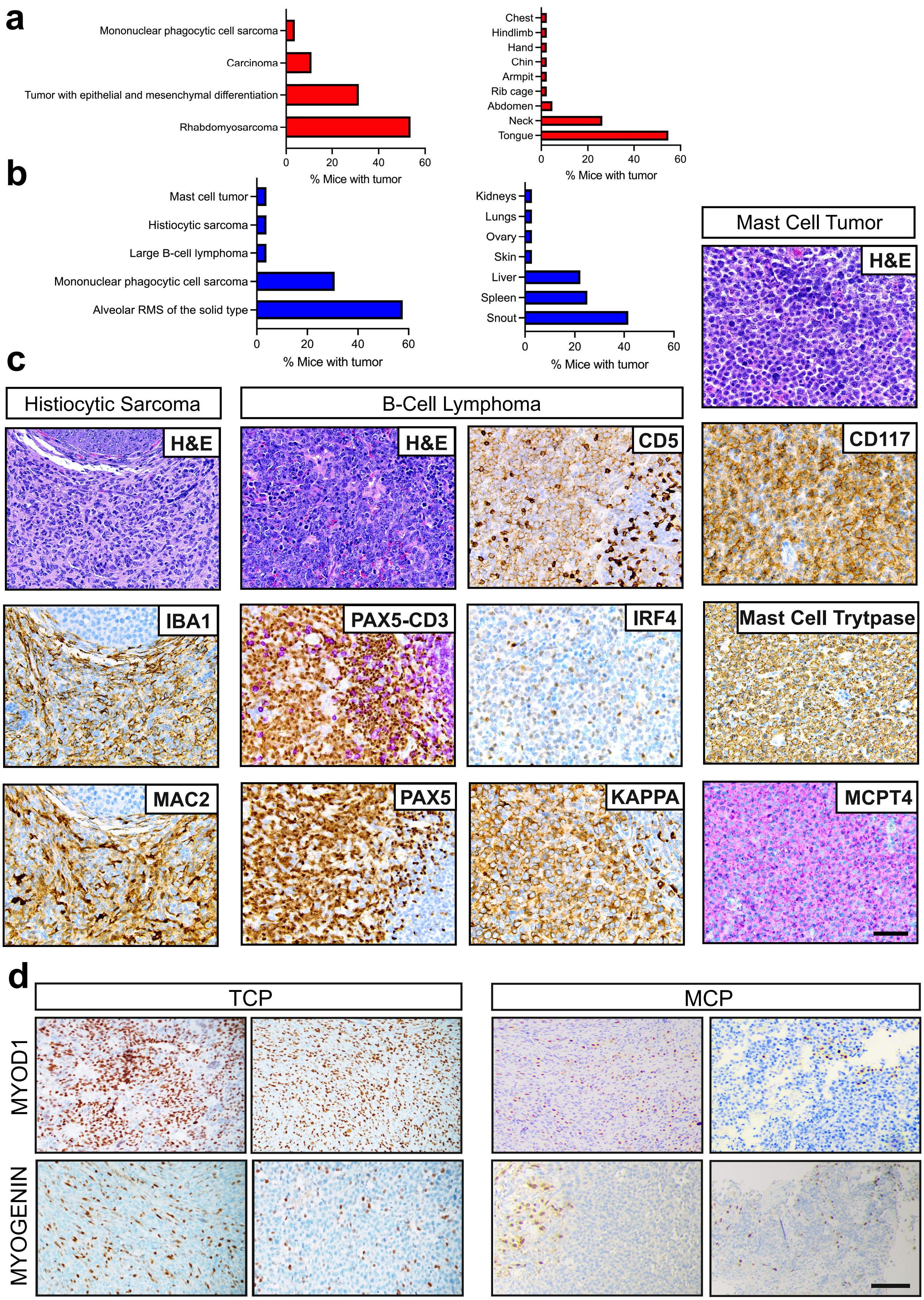

Supplementary Figure S4_1 Searcy and Larsen et al.

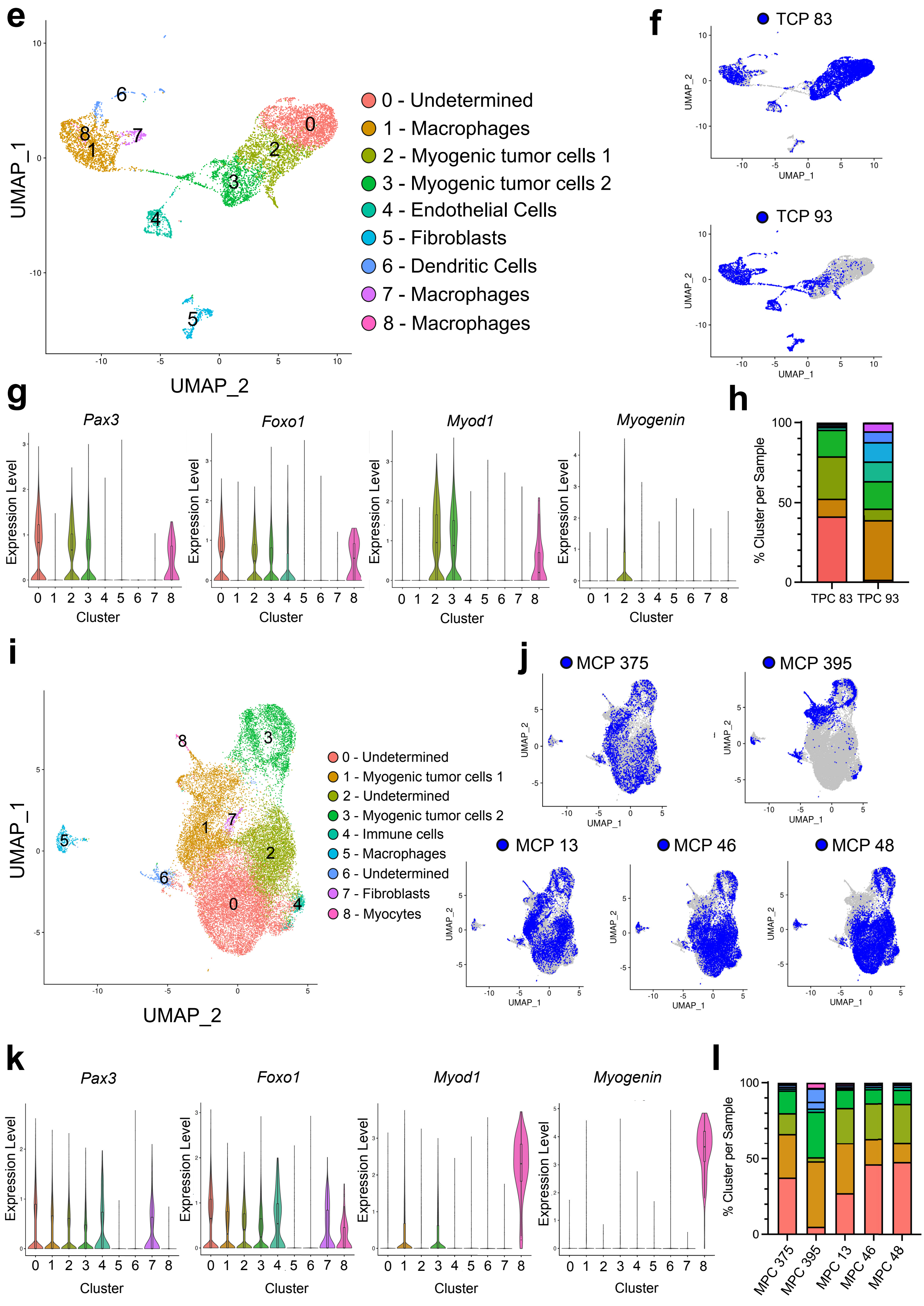

Supplementary Figure 4_2 Searcy and Larsen et al.

Supplementary Figure 4. TCP and MCP develop multiple tumor types. **a** TCP tumor types (left), and tumor locations (right). **b** MCP tumor types (left), and tumor locations (right). **c** IHC for diagnostic staining for B-Cell Lymphoma (H&E, PAX5-CD3, PAX5, CD5, IRF4 and KAPPA) (n = 1 mouse), Histiocytic Sarcoma and Mononuclear Phagocytic Cell Sarcoma (H&E, IBA1 and MAC2)(n = 11 mice), and Mast Cell Tumor (H&E, CD117, Mast Cell Tryptase and MCPT4)(n = 1 mouse). Scale Bar = 50μm. **d** IHC for MYOD1 and MYOGENIN staining on TCP and MCP tumors (n = 2 tumors per genotype). Scale bar = 100 μm. **e** UMAP and cluster analysis of single-cell transcriptional profiles of two separate TCP tumors. **f** UMAP highlighted by sample. **g** Violin plots showing expression of *Pax3, Foxo1, Myod1,* and *Myogenin* in each cluster. The median is defined as the 50% quantile. The upper whisker represents the largest observation ≤ to the upper hinge (75% quantile) +1.5*IQR, where the IQR is the interquartile range, or the distance between the first and third quartiles. The lower whisker represents the smallest observation ≥ to the lower hinge (25% quantile) -1.5*IQR. **h** Proportions of clusters present two TCP tumors. **i** UMAP and cluster analysis of single-cell transcriptional profiles of five separate MCP tumors. **j** UMAP highlighted by sample. **k** Violin plots showing expression of *Pax3, Foxo1, Myod1,* and *Myogenin* in each cluster. The median is defined as the 50% quantile. The upper whisker represents the largest observation ≤ to the upper hinge (75% quantile) +1.5*IQR, where the IQR is the interquartile range, or the distance between the first and third quartiles. The lower whisker represents the smallest observation ≥ to the lower hinge (25% quantile) -1.5*IQR. **l** Proportions of clusters present five MCP tumors. Source data are provided as a Source Data file.

a

Cell State Signature	# of Genes Mouse	# of Genes Human
Apoptosis	56	80
Hypoxia	42	42
Interferon	38	46
Mesenchymal-like	125	133
Muscle	68	75
Proliferation	112	114

b

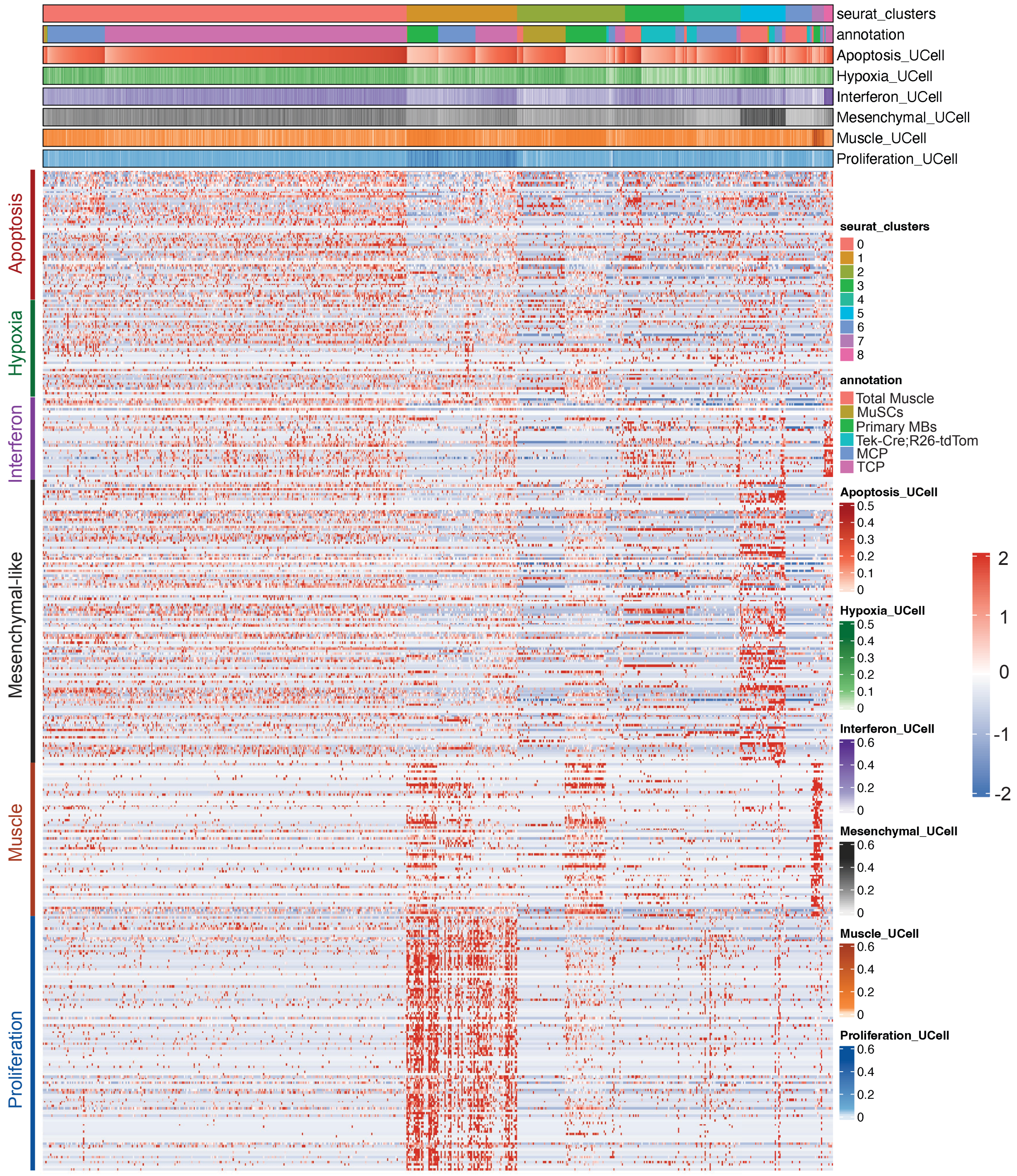

Supplementary Figure 5 Searcy and Larsen et al.

Supplementary Figure 5. Extended comparison of human RMS gene modules in mouse RMS. **a** Table showing how many genes from the complete list of genes that define human RMS transcriptional modules derived from Wei et al.[43] could be converted to mouse based on orthology. **b** scRNAseq gene expression of all genes from the cell state signature gene modules identified in human RMS PDXs (Proliferation, Muscle, Hypoxia, Mesenchymal-like and Apoptosis) derived from Wei et al.[43] in mouse MCP and TCP tumors, primary myoblasts, muscle stem cells, total muscle, and Tom^{+} cells from *Tek-Cre;R26-tdTomato* muscle. Heatmap illustrates single cells from mouse samples (x-axis) and the normalized gene expression in cell state signature gene modules (y-axis). UCell signature score[104] is calculated for every module and visualized in the color bar above the heatmap.

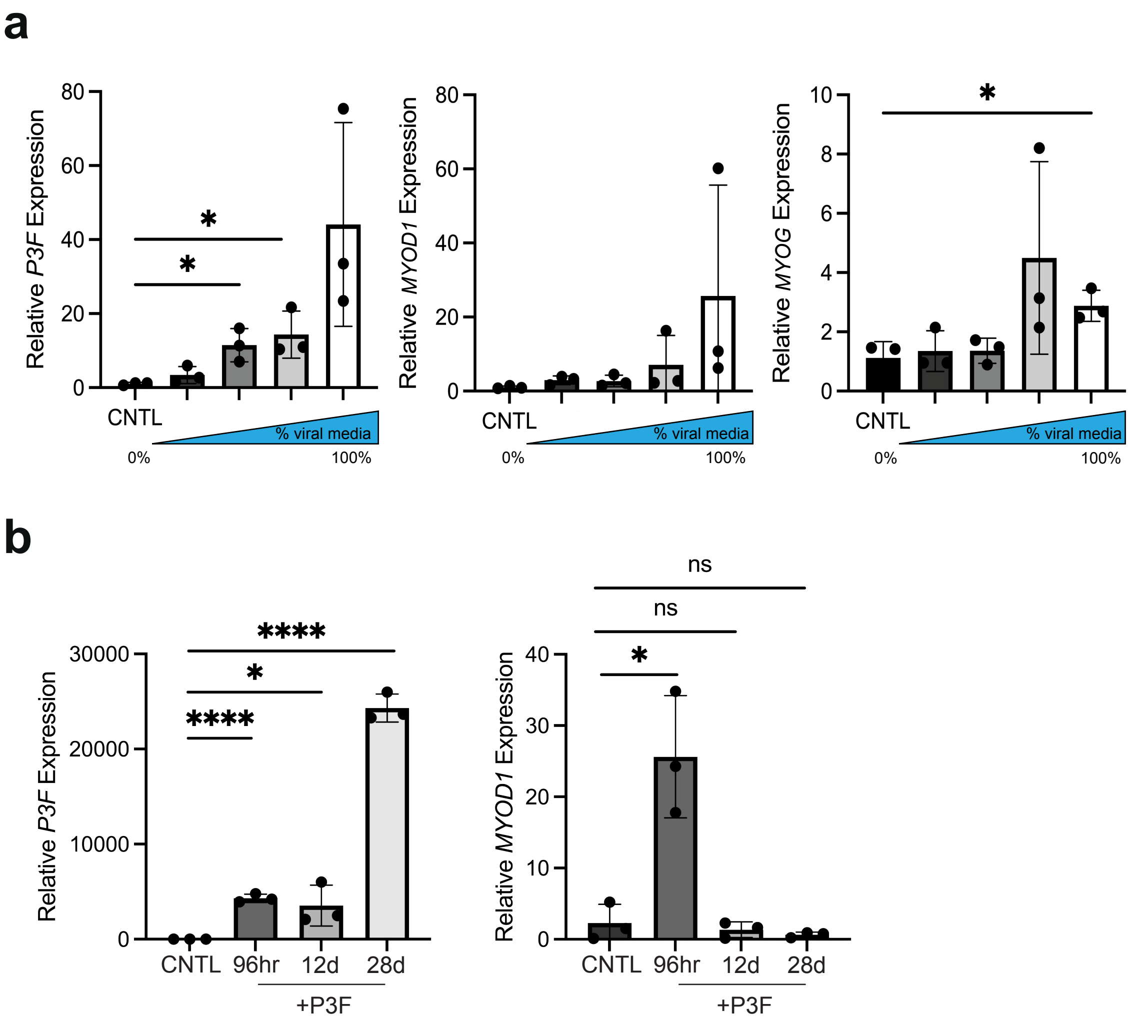

Supplementary Figure 6. P3F expression in WT HUVECs induces myogenic gene expression. **a** Gene expression by real time PCR for *P3F* (“50%” P = 0.0159, “75%” P = 0.0227), *MYOD1*, and *MYOG* with 25%, 50%, 75%, and 100% virus (visualized by increasing blue triangle below the graph) and not transduced cells as CNTL (n = 3 technical replicates). Only statistically significant differences are noted. **b** Gene expression by real time PCR for *P3F* (“96 hr” P < 0.0001, “12 d” P = 0.0465, “28 d” P < 0.0001) and *MYOD1* (“96 hr” P = 0.0108, “12 d” P = 0.6015, “28 d” P = 0.3432) at three time points after transduction with P3F (n = 3 technical replicates). All P values in pairwise comparisons were determined by Student’s *t*-test (unpaired, two-tailed); *P < 0.05, ****P < 0.0001. Data represented as mean +/- SEM. Source data are provided as a Source Data file.

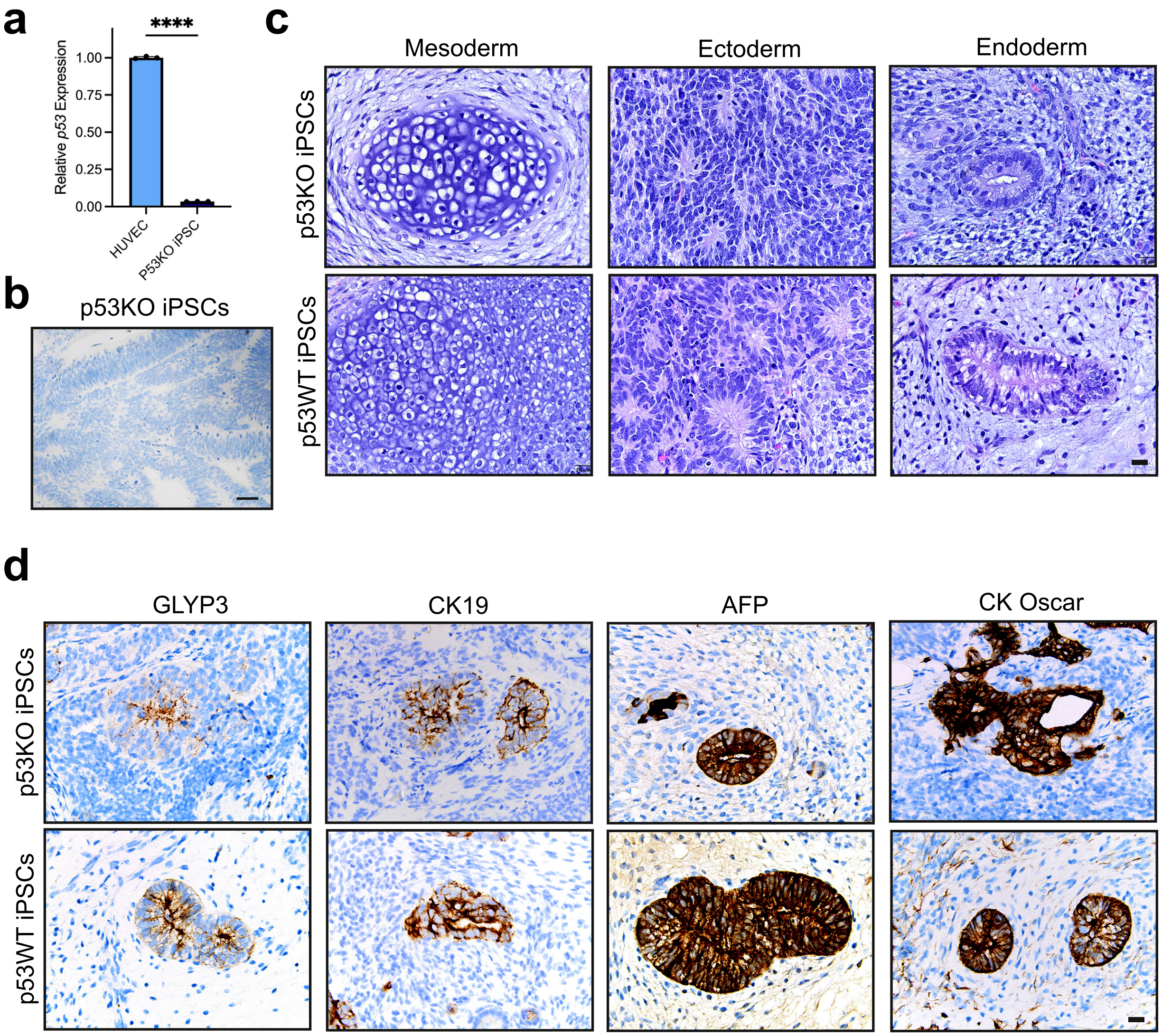

Supplementary Figure 7. Comparison of *TP53* WT and KO human iPSC derived teratomas. **a** Gene expression by real time PCR for *TP53* in $p53^{KO}$ iPSCs compared to HUVECs (n = 3 technical replicates). **b** Representative histology of $p53^{KO}$ teratomas stained for TP53 (n = 3 separate teratomas). Scale bar = 100 µm. 3. **c** Representative histology of $p53^{KO}$ (n = 3 tumors) and $p53^{WT}$ (n = 5 tumors) iPSC teratomas stained for H&E (n = 3 tumors per genotype). Mesodermal derived cartilage (left), neuroectodermal rosettes (middle), endodermal derived glands (left). Scale bar = 20 µm. **d** Representative histology of p53KO and p53WT iPSC teratomas stained for GLYP3, CK19, AFP and CK Oscar (n = 3 per tumors per genotype). Scale bar = 20 µm. All P values in pairwise comparisons were determined by Student's *t*-test (unpaired, two-tailed); ****P < 0.0001. Source data are provided as a Source Data file.

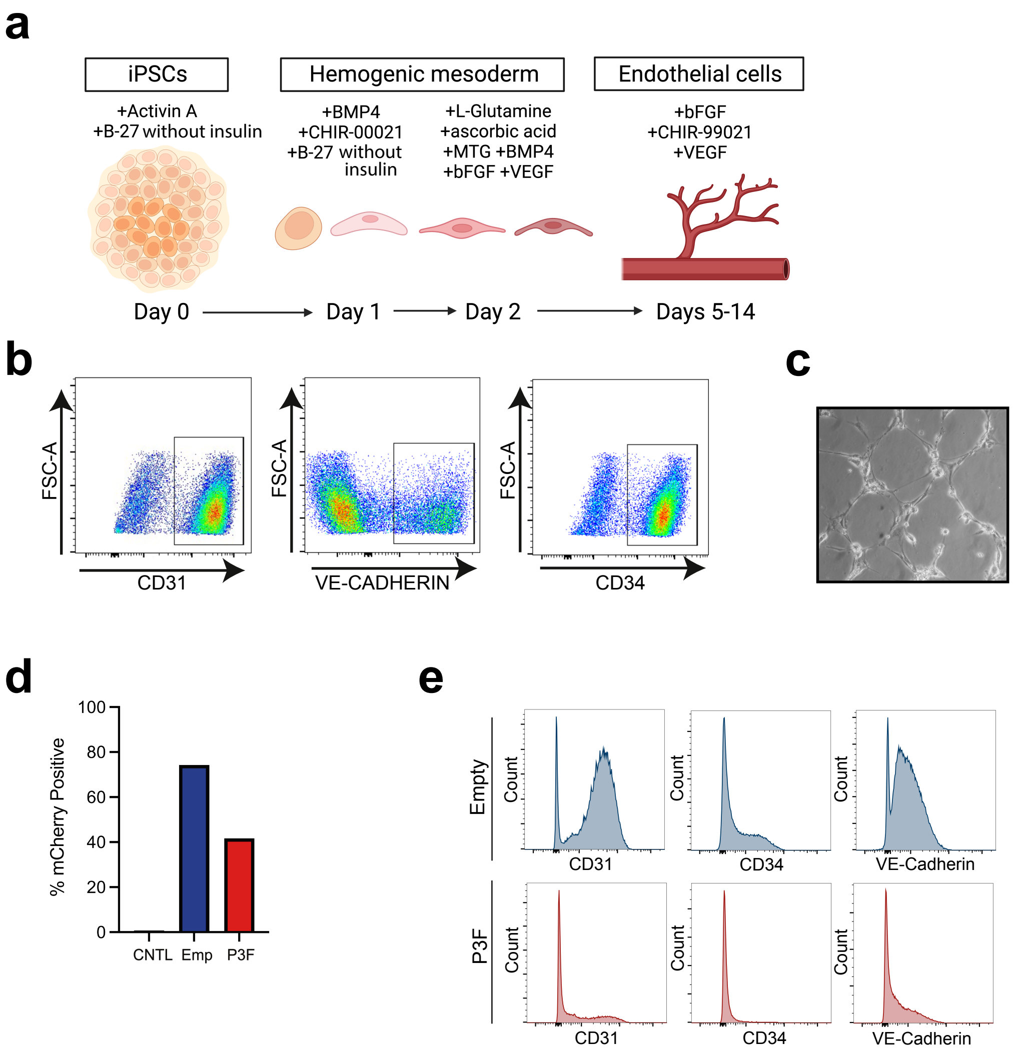

Supplementary Figure 8. Endothelial differentiation of human iPSCs and transduction with P3F. **a** Schematic of 14-day endothelial differentiation protocol with respective growth factors added at each step of the protocol. Created with BioRender.com. **b** Representative flow cytometry analysis confirming the expression of endothelial markers, CD31, VE-CADHERIN, and CD34 on iPSCs differentiated to ECs (n = 3). **c** EC tube formation assay on iPSCs differentiated to ECs (n = 3). **d, e** Quantification of flow cytometry analysis for mCherry, CD31, CD34, and VE-Cadherin in P3F transduced cells. Source data are provided as a Source Data file.

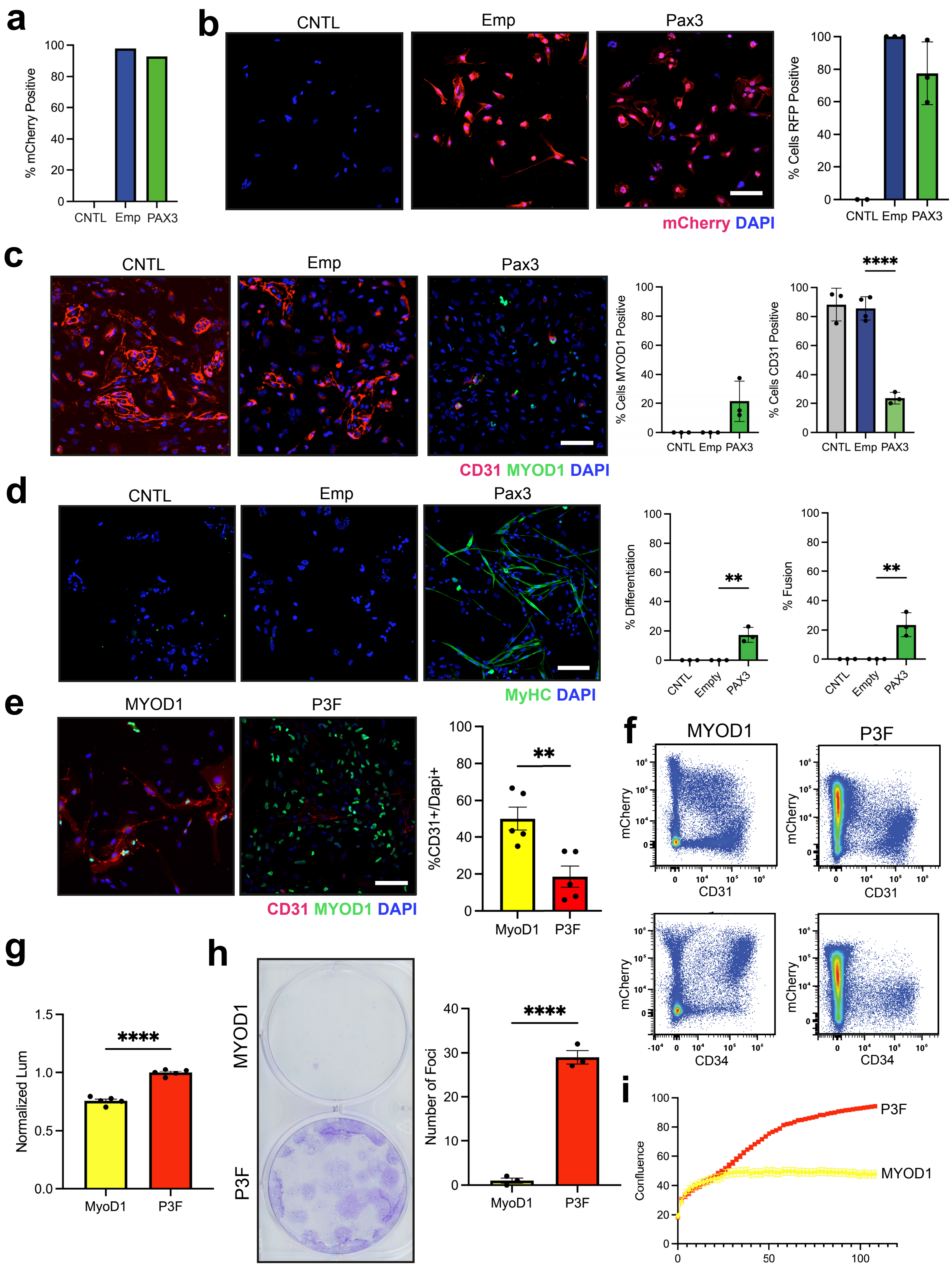

Supplementary Figure 9: Searcy and Larsen et al.

Supplementary Figure 9. PAX3 and MYOD1 are not sufficient to induce myogenic reprogramming and tumorigenesis of endothelial differentiating cells. **a-i** Representative analysis from one experimental replicate. Data collected at the end of the 14-day endothelial differentiation protocol. "Emp" cells are $p53^{KO}$ iPSCs transduced with pSin-mCherry and "CNTL" (control) cells are untransduced $p53^{KO}$ iPSCs (n = 3 experimental replicates). **a** Quantification of flow cytometry analysis for mCherry expression in Lenti-*PAX*3 transduced cells compared to CNTL and empty. **b** Representative IF for mCherry (red) in Lenti-*PAX*3 transduced cells compared to CNTL and empty. DAPI nuclear stain (blue). Scale bar = 50 μm. Quantitation of %RFP+ cells/Total cells shown (Emp and PAX3 n = 3 experimental replicates per group, CNTL n = 2 experimental replicates). **c** Representative IF for MYOD1 (green), CD31 (red), DAPI nuclear stain (blue) in Lenti-*PAX*3 transduced cells compared to CNTL and empty. Scale bar = 50 μm. Quantitation of %MYOD1+ cells/Total cells (n = 3) and %CD31+ cells/Total cells shown (CNTL and PAX3 n = 3, Emp n = 3). **d** Representative IF of myogenic differentiation assay for MyHC (green) showing differentiated myotubes in Lenti-*PAX*3 transduced cells compared to CNTL and empty. DAPI nuclear stain (blue). Scale bar = 50 μm. Quantitation of differentiated myotubes (%MyHC+ cells/Total cells) (n = 3 fields of view, P = 0.0043) and fused myotubes (%Nuclei in multinucleated MyHC+ tubes/Total nuclei) (n = 3 technical replicates, P = 0.0079) shown. **e** Representative IF for MYOD1 (green), CD31 (red), DAPI nuclear stain (blue) in Lenti-*MYOD1* transduced cells compared to Lenti-*P3F* transduced cells. Scale bar = 50 μm. Quantitation of %CD31+ cells/Total cells is shown (n = 5 fields of view, P = 0.0062). **f** Representative flow cytometry for CD31 and CD34 in Lenti-*MYOD1* transduced cells compared to Lenti-*P3F* transduced cells. **g** Relative viability by Cell Titer Glow assay comparing lenti-*MYOD1* transduced cells compared to lenti-*P3F* transduced cells after 48 hours (normalized to P3F, n = 3 technical replicates, P <0.0001). **h** Representative well from low density focus formation assay stained with crystal violet in lenti-*MYOD1* transduced cells compared to lenti-*P3F* transduced cells. Quantitation of foci/well shown (n = 3 wells, P <0.0001). **i** Incucyte proliferation assay comparing time (x-axis) to reach confluence (y-axis) in Lenti-*MYOD1* transduced cells compared to Lenti-*P3F* transduced cells. All P values in pairwise comparisons were determined by Student's *t*-test (unpaired, two-tailed); **P < 0.01, ****P < 0.0001. Data represented as mean +/- SEM. Source data are provided as a Source Data file.

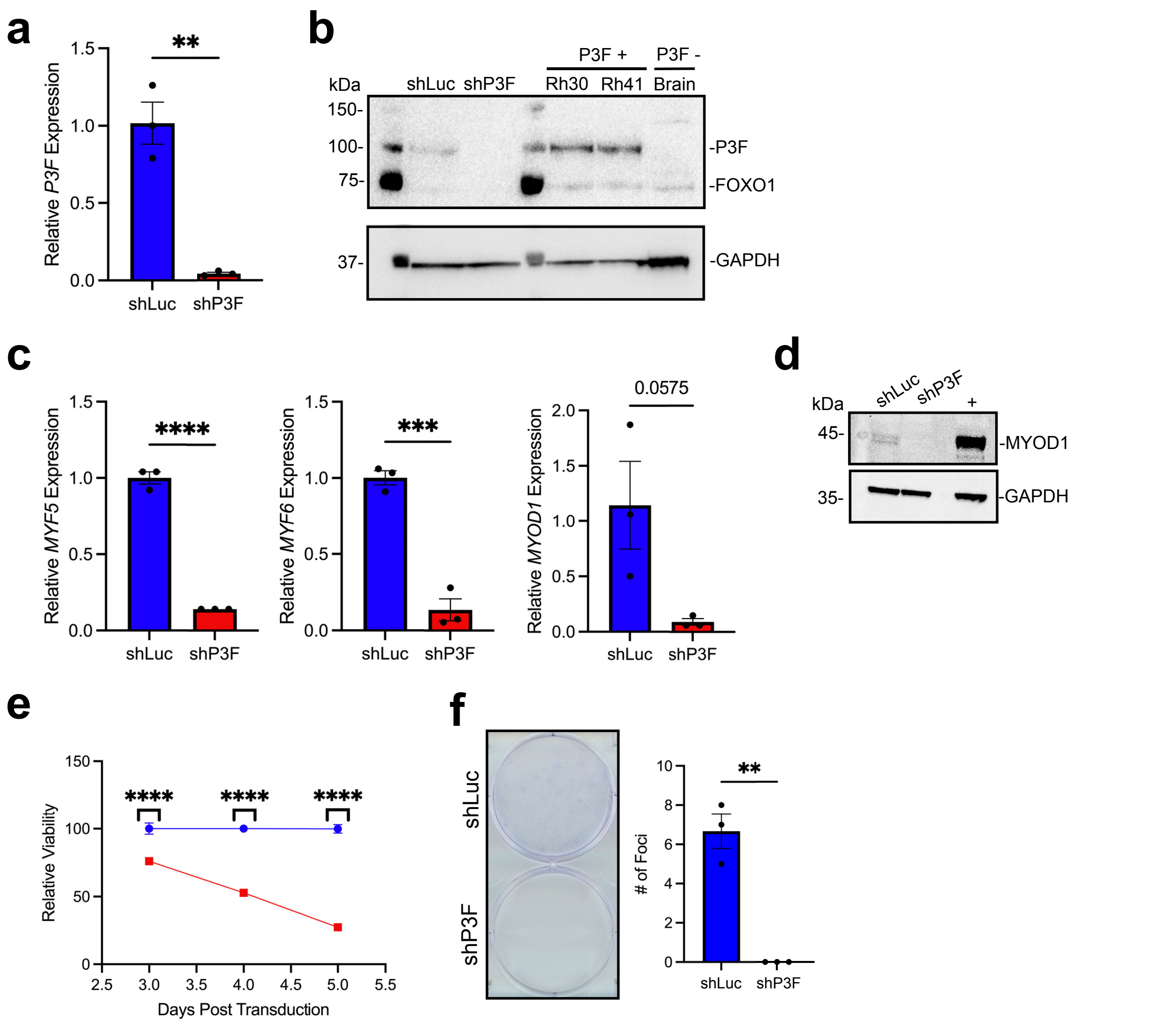

Supplementary Figure 10. P3F expression is required to maintain myogenic and tumorigenic identity of FP-RMS cells. **a** Gene expression by real time PCR for *P3F* in transformed P3F expressing p53^{KO} iPSCs transduced with control shRNA (shLuc) and shRNA targeting P3F (shP3F) (n = 3 technical replicates, P = 0.002). **b** Immunoblot of FOXO1 detecting FOXO1 and P3F, and GAPDH in pLKO-shLuc and pLKO-shP3F transduced cells. **c** Gene expression by real time PCR for *MYF5* (P <0.0001)*, MYF6* (P = 0.0005)*,* and *MYOD1* (P = 0.0575) in pLKO-shLuc and pLKO-shPF1 transduced cells (n = 3 technical replicates). **d** Immunoblot for MYOD1 and GAPDH in pLKO-shLuc and pLKO-shP3F transduced cells. **e** Relative viability measured by CellTiter-Glo (CTG). Timepoints taken at 3 (P = 0.000844), 4 (P < 0.000001), and 5 (P < 0.00001) days post transduction with pLKO-shRNA (n = 5 technical replicates). **f** Low density focus formation assay performed on pLKO-shLuc and pLKO-shP3F transduced cells (n = 3 wells, P = 0.0016). All P values in pairwise comparisons were determined by Student's *t*-test (unpaired, two-tailed); *P < 0.05, **P < 0.01, ***P < 0.001, ****P < 0.0001. Data represented as mean +/- SEM. Source data are provided as a Source Data file.

Supplementary Table 1. List of primers used for quantitative real-time PCR

Gene	Species	Right Primer	Left Primer
Pax3-Foxo1	Mouse	ACAGACAGCTTTGTGCCTCC	GAAGGGACAGATTGTGGCGAA
MyoD1	Mouse	GTCGTAGCCATTCTGCCG	AGCACTACAGTGGCGACTCA
MyoG	Mouse	GTGGGAGTTGCATTCACTGG	CTACAGGCCTTGCTCAGCTC
Tomato		AGTTCATGTACGGCTCCAAGG	AGCGCGTGATGAACTTCGA
PAX3-FOXO1	Human	TCCAACCCCATGAACCCC	GCCATTTGGAAAACTGTGATCC
MYOD1	Human	GGGCGCCTCGTTGTAGT	AACTGCTCCGACGGCAT
MYOG	Human	GCTGTGAGAGCTGCATTCG	CAGCTCCCTCAACCAGGAG
PECAM1	Human	ATCATTTCTAGCGCATGGCCTGGT	ATTTGTGGAGGGCGAGGTCATAGA
CD34	Human	AAATCCTCTTCCTCTGAGGCTGGA	AAGAGGCAGCTGGTGATAAGGGTT
VE-CADHERIN	Human	CCTCTCAATGGCGAACAC	ATGTAGGCAAGATCAAGTCAAG
MYF5	Human	TCAGGACAGTAGATGCTGTCAAA	CACCTCCAACTGCTCTGATG
MYF6	Human	GGAGCGCCATCAGCTATATTG	ATCCGCACCCTCAAGATTTTC
