# Supplementary material for: PAX3-FOXO1 dictates myogenic reprogramming and rhabdomyosarcoma identity in endothelial progenitors
Source: Nat Commun. 2023 Nov 15;14:7291. doi: 10.1038/s41467-023-43044-1 (PMC10651858; doi:10.1038/s41467-023-43044-1)
Supplement: Supplementary file 31 — Reporting Summary [file 41467_2023_43044_MOESM31_ESM.pdf]

nature portfolio

Corresponding author(s): Mark Hatley

Last updated by author(s): Oct 8, 2023

Reporting Summary

Nature Portfolio wishes to improve the reproducibility of the work that we publish. This form provides structure for consistency and transparency in reporting. For further information on Nature Portfolio policies, see our Editorial Policies and the Editorial Policy Checklist.

Please do not complete any field with "not applicable" or n/a. Refer to the help text for what text to use if an item is not relevant to your study.
For final submission: please carefully check your responses for accuracy; you will not be able to make changes later.

Statistics

For all statistical analyses, confirm that the following items are present in the figure legend, table legend, main text, or Methods section.

n/a	Confirmed	
☐	☒	The exact sample size (*n*) for each experimental group/condition, given as a discrete number and unit of measurement
☐	☒	A statement on whether measurements were taken from distinct samples or whether the same sample was measured repeatedly
☐	☒	The statistical test(s) used AND whether they are one- or two-sided<br>*Only common tests should be described solely by name; describe more complex techniques in the Methods section.*
☐	☒	A description of all covariates tested
☐	☒	A description of any assumptions or corrections, such as tests of normality and adjustment for multiple comparisons
☐	☒	A full description of the statistical parameters including central tendency (e.g. means) or other basic estimates (e.g. regression coefficient) AND variation (e.g. standard deviation) or associated estimates of uncertainty (e.g. confidence intervals)
☐	☒	For null hypothesis testing, the test statistic (e.g. F, t, r) with confidence intervals, effect sizes, degrees of freedom and P value noted<br>*Give P values as exact values whenever suitable.*
☒	☐	For Bayesian analysis, information on the choice of priors and Markov chain Monte Carlo settings
☐	☒	For hierarchical and complex designs, identification of the appropriate level for tests and full reporting of outcomes
☒	☐	Estimates of effect sizes (e.g. Cohen's *d*, Pearson's *r*), indicating how they were calculated

Our web collection on statistics for biologists contains articles on many of the points above.

Software and code

Policy information about availability of computer code

Data collection

Images captured on a Nikon Eclipse 80i upright fluorescent microscope or a Leica DMi 8 Thunder Imager inverted fluorescent microscope.

Muscle was dissected and digested then stained for flow cytometry prior to analysis with a BD Biosciences Symphony analyzer for flow cytometry analysis and a BD Biosciences Aria cell sorter for cell sorting. Flow cytometry analysis for endothelial differentiation markers shown in Supplementary Figure 9f was performed on a Cytek Aurora spectral analyzer

Total RNA was isolated from tissues or cells using miRNEasy mini kit (#217004, Qiagen) and from sorted cell populations using a miRNEasy micro kit (#217084, Qiagen) according to the manufacturer's instructions. Superscript III First Strand Synthesis using random hexamer primers (#18080051, ThermoFisher) was used to synthesize first-strand cDNA. SYBR primers were used for qPCR. RNA sequencing was performed on purified RNA using the Illumina NovaSeq6000 genome sequencing system.

Mononuclear cells were dissociated from muscle or tumor and live tomato-positive cells collected by FACS and loaded on the 10x Chromium platform and processed using manufacturers protocol for cDNA synthesis and library preparation. CUT&RUN was performed using the CUTANA ChIC/CUT&RUN kit (14-1048, EpiCypher) and each reaction used 250,000 nuclei. Libraries were prepared with the xGen ssDNA & Low-Input DNA Library Prep Kit (#10009859, IDT). HiC and library preparation was performed on 500,000 cells per replicate using reagents and protocol from the Arima-HiC Kit (#A510008, Arima). For RNAseq, CUT&RUN, and HiC, quality checks were performed using the Agilent 4150 TapeStation and Agilent Bioanalyzer before sequencing with NovaSeq6000 (Illumina).

Cells assessed for viability with CellTiterGlo (#G7570, Promega). CellTiterGlo assays performed according to manufacturer's protocol; luminescence measured on a BioTek Synergy 2 and BioTek's Gen5 1.11 software.

Data analysis

Flow cytometry data analyzed with FlowJo 10.8.1. and SpectroFlo 3.0.3. (Supp Fig 9F).

Bulk RNA-seq data analysis - Mouse and human sequences were mapped to the mm10 and hg38 genomes, respectively, using the STAR aligner. Gene level quantification was determined using RSEM70 and based on GENCODE annotations M22 (for mouse samples) or Human Release 31 (for human samples), respectively. Non-coding and GENCODE level 3 genes were excluded. Differential gene expression was modeled using the voom method, available in the Limma R software package. Normalization factors were generated using the TMM method. Voom normalized counts were analyzed using the lmFit and eBayes functions in Limma. The false discovery rate (FDR) was estimated using the Benjamini-Hochberg method. Gene set enrichment analysis (GSEA) was performed using curated signatures from MSigDB. Ranking of genes was calculated using negative log10(p-value)*log2(fold change), and a P-value for each gene set was estimated by comparing the observed enrichment score to that obtained from a null distribution computed from 1000 permutations of genes within gene sets. FDR was estimated as previously described. Functional annotation clustering by the Database for Annotation, Visualization, and Integrated Discovery (DAVID, 2021 version) was used for gene ontology (GO) analyses. We used GOTERM_BP_FAT selection for functional annotation clustering and present the top ten unique GO terms visualized and represented as the -log10Pvalue.
Single-Cell RNA-Seq Analysis - We used Cell Ranger v.4.0.1 pipeline (10x Genomics) with default parameters to aligned raw reads to the GRCm38 Ensembl 97 genome (mm10) and to generate raw gene-barcode matrices. We use Seurat (version 4.0.6, R version 4.1.0) to perform merging, thresholding, normalization, principal component analysis, non-linear multidimensional reduction, clustering analysis, visualization and differential gene expression analysis. Quality filtering was conducted by excluding low-quality or doublet outlier cells with values beyond 3x Median Absolute Deviation (MAD) from the median in the QC metrics of nFeature_RNA, nCount_RNA or mitochondria count per cell and removing genes expressed in less than 3 cells. To identify the integration of anchor genes among the samples, the FindIntegrationAnchors function was used with default parameters. Using Seurat's IntegrateData function, samples were combined into one object. Data were normalized using the SCTransform function and then principal component analysis (PCA) was used to reduce the dimensionality of this dataset. The number of relevant dimensions was determined by ElbowPlot function, and these PCs were summarized further using Uniform Manifold Approximation and Projection (UMAP). For cell clustering, we used highly variable genes selected using FindVariableFeatures function with default parameters. In addition, nCount_RNA and percentage of mitochondrial genes were considered as the source of unwanted variability and were regressed out. Clustering was conducted using the FindNeighbors and FindClusters functions with original Louvain algorithm and a resolution parameter of 0.2. Differential gene expression analysis was performed by the FindAllMarkers function in Seurat. Clusters were annotated using markers genes found in the literature in combination with differentially expressed genes. We use Seurat methods (DimPlot, FeaturePlot, VlnPlot, DoHeatmap etc.) or custom functions for data visualizations including UMAP, feature plot, violin plot and heatmap. Additional Note: Due to suboptimal QC metrics in some samples/analyses, performed additional manual cell filtering with arbitrary cutoffs. Supplementary Figure. 1j: CAB-2829, retain cells with minimum 1000 detectable genes per cell and at least 2500 UMI counts per cell. Supplemenatry Figure. 1m: CAB-3347, retain cells with minimum 1000 detectable genes per cell per cell. Cell dynamics were analyzed using scVelo (version 0.2.4) package implementation in Scanpy (version 1.7.2). The spliced / unspliced count matrix was generated by Velocyto (version 0.17.17) and was import to Scanpy_AnnData object. Then cell barcodes, clusters and UMAP embeddings were imported from Seurat object. Then the data was then processed using default parameters following Scanpy scVelo implementation. In short, the samples were pre-processed using scVelo functions for filtering and normalization using scv.pp.filter_and_normalise and followed by scv.pp.moments function. We used scv.tl.recover_dynamics function to recover the full splicing kinetics of specified genes. The gene specific velocities were then calculated using scv.tl.velocity with dynamic mode, and scv.tl.velocity_graph functions and visualized using scv.pl.velocity_graph function. For Partition-based graph abstraction (PAGA) analysis, we used Scanpy scVelo implementation function scv.tl.paga with default parameters and scv.pl.paga function for velocity-driven PAGA analysis and plotting PAGA graph with velocity-directed edges.
CUT&RUN analysis - Paired-end CUT&RUN-seq reads were aligned first to the E. coli reference genome using bowtie(version 1.2.2) in pairedend
with parameters -k 1 –best and –un to retain unmapped reads. Because of nonbiological variability among samples, the numbers of E. coli reads were not used for cell-number-normalization hereafter. Retained non-E. coli reads were then mapped to the mouse reference genome (mm10) or human reference genome (hg38) using bowtie in paired-end mode with parameters -p 20 -k 2 -m 2 –best. A BAM file containing CUT&RUN-seq fragments was computationally created from each aligned read-pair using samtools sort -n, bedtools bamToBed -bedpe, a manual conversion from BEDPE to BED3, and bedtools bedToBam. For display, the reference genomes were binned into 50bp windows using bedtools makewindows, and coverage of those bins by fragments was calculated using bedtools intersect -c. Fragment coverage was normalized per million mapped fragments, converted to bedGraph using bedGraphToBigWig, converted to wiggle using bigWigToWig, converted to TDF using igvtools toTDF, and then visualized in the IGV(version 2.16.2.) browser in hg38 for human samples.
MACS1.4 was used to identify regions significantly enriched in CUT&RUN-seq reads. Prior to peak-calling, individual reads from each pairedend
alignment were discarded if they overlapped the ENCODE-defined Problematic Regions list corresponding to the reference genome in use, and further filtered in mouse if they overlapped the region chr1:24611436-24616256, because of an observed genomically amplified region in all samples near the Col19a1 gene. Retained reads were used for peak-calling against each sample's corresponding, identically processed, control IgG. In mouse, parameters –keep-dup=auto and –p 0.001 were used; in human, parameters –keep-dup=auto and -p 1e-9 were used. To compare TCP vs. MCP coverage at regions of interest, we collapsed peaks called separately in each sample (N=3 TCP and N=4 MCP in mouse) using bedtools merge. Bedtools closest -t first was used to annotate each peak with the name of the gene whose transcript has the nearest transcription start site from mm10_refGene.gtf downloaded from the UCSC Genome Browser. Individual read coverage in each of the seven samples was separately quantified using bedtools intersect -c. For comparative analyses (Fig.5c), read coverage in each sample was normalized to the millions of mapped reads (RPM), and the average RPMs from each condition were calculated. For statistical analysis (Fig. 5d) of differential coverage, raw reads from the three TCP samples and three representative MCP samples (MCP20_1, MCP20_2, and MCP_38) were used as input for DEseq2. Peaks were considered differentially covered if they had DEseq2 adjusted log2 fold-change >1 or <-1 and adjusted p value of 0.05.
We used ROSE51 (https://bitbucket.org/young_computation/rose) to separately identify super-enhancers from each sample of MCP, TCP, Day 15 Untransduced p53KO iPSC, and Day 15 Lenti-P3F transduced p53KO iPSC CUT&RUN-seq data targeting H3K27ac, as previously described. Each sample was separately processed with its corresponding IgG. For each sample, we used MACS1.4 to identify two sets of peaks from each read set filtered as above. In mouse, we used parameter sets –keep-dup=auto –p 0.001 and –keep-dup=all –p 0.001; in human, we used parameter sets –keep-dup=auto -p 1e-9 and –keep-dup=all -p 1e-9. These two peak sets from each sample were collapsed into a per-sample set of peaks using bedtools merge; these collapsed peaks were used as input for ROSE with parameters -s 12500 -t 1000.
nature portfolio | reporting summary March 2021
Regions displayed in Figures 5e and f were created from the collapsed union of super-enhancers for MCP (n = 4) and TCP (n = 3). Coverage of these collapsed regions in each condition was separately quantified using bedtools intersect -c. For comparative analyses, we followed the same strategy described in peak-based comparative analyses to normalize read coverage in each sample to the millions of mapped reads (RPM), and the average RPMs from each condition were calculated. The DESeq2 package and its standard parameters was used to identify

statistically differential coverage of super-enhancers between TCP and MCP replicates, by using three TCP samples and three representative MCP samples (MCP20_1, MCP20_2, and MCP_38). Significantly differentially covered super-enhancers met two thresholds: adjusted Pvalue= 0.05 and absolute log2Fold change (MCP/TCP)>1. After collapsing, super-enhancers were assigned to the single gene with a transcript whose start site from the mm10_refGene.gtf gene list is nearest the center of the collapsed super-enhancer using bedtools closest.
To determine if enhancers or super-enhancers were specifically activated to drive specific gene sets of known function, we performed Gene Set Enrichment Analysis (GSEA). DESeq2 log2FC(MCP/TCP) tables associated with collapsed enhancers (Fig. 5d) or collapsed super-enhancers (Fig. 5f) were used as raw table for preparing inputs for GSEA Preranked (version 4.3.2). Each enhancer/super-enhancer is associated with a gene, so a single gene could have multiple ranked loci associated with it on the raw DESeq2 log2FC(MCP/TCP) tables mentioned above. The single representative enhancer/super-enhancer for each gene was selected as the one with the highest absolute DESeq2 log2FC(MCP/TCP). These ranked genes were analyzed for their enrichment using the Gene Ontology hallmarks collection in MSigDB. We used parameters minSize=15, maxSize = 500, nperm=1000 for collapsed peaks GSEA Preranked analysis, and minSize=1, maxSize = 2500, nperm=1000 for preranked
log2FC(MCP/TCP) from collapsed Super-Enhancers GSEA Preranked analysis.
Motif enrichment analysis - We performed a motif enrichment analysis in putative enhancers using AME (Analysis of Motif Enrichment). A list of transcription factors associated with the endothelial lineage was built from literature analysis and is available as (Supplementary Table 11). We selected the Mus musculus-sourced endothelial TF motifs PWMs (Position-Weight Matrix) from the CIS-BP 2.00 database94 corresponding to these TFs. Coordinates of putative enhancers were defined as above using CUT&RUN-Seq from MCP or TCP cells with only the parameter set -p 0.001 –keep-dup=auto. For each transcript from mm10_refGene.gtf, its putative promoter region was defined as +/- 2kb from the TSS (Transcription Start Site); we used bedtools subtract to remove sections of H3K27ac peaks defined as above that overlap these promoters because H3K27ac marks both active promoters and enhancers. The sequences of these remaining regions were acquired using bedtools getfasta and the mm10 reference genome. AME was then used to identify the statistically enriched TF motif hits within the sequences of these putative enhancer regions, with the command 'ame --control --shuffle method fisher'.
HiC Analysis - For display of HiC interaction frequencies, paired-end reads of 150 bp were processed by Juicer (v1.5, default parameters) based on mm10 and Arima fragmentation (cut sites GATC or GANTC). Each replicate was processed separately using default parameters (every replicate got > 500M pairs as > 360M contacts). We required MAPQ score greater than 30 for generating .hic file and downstream analysis. Reproducibility have been confirmed by visual inspection.
For genome-wide comparisons of contact frequencies, we applied HiC-Pro (v 2.11.1)96 to perform read alignment, read filtering, quality check, and contact matrix building as described with default parameters unless otherwise specified below. The two replicate paired-end FASTQ files from each condition were separately pooled, and to achieve improved performance, we split these pooled FASTQ files into multiple subsets, each with a maximum of 10 million read pairs, which were used as input for HiC-Pro in parallel mode. HiC-Pro was configured so that each end of the read pair was mapped independently to the mm10 reference genome using bowtie2 (v2.3.5.1). For global and local alignment, we used the options “bowtie2 --very-sensitive -L 30 --score-min L,-0.6,-0.2 --end-to-end –reorder” and “bowtie2 --verysensitive
-L 20 --score-min L,-0.6,-0.2 --end-to-end –reorder” respectively. For read mapping, we used MAPQ threshold=10 and removed multi-mapped reads (RM_MULTI=1) and duplicated read pairs (RM_DUP=1). Taking the final valid read pairs generated from HiC-Pro as input, we used the “hicpro2juicebox.sh” utility script to create a HiChIP contact map in “.hic” format. The bin intervals files fastq.matrix from HiC-Pro outputs were combined as RelevantBins matrices between TCP and MCP at 5Kb, 10Kb, 100Kb, and 1Mb bin sizes. The TCP and MCP connectivity RelevantBins combined matrices were used for generating the scatterplot of TCP and MCP in Figure 5a.
Scatterplots comparing MCP vs. TCP connectivity were assembled from aligned HiC data utilizing the ggplot2 package in R. All bin-pairs with at least one read connecting them were used as an input for a matrix that describes connectivity across experiments. Reads connecting pairs of bins (bin-pairs) were plotted. A subset of points was highlighted if either of the bins comprising a bin-pair overlapped a preselected gene of interest by at least 1bp. Hoxa9 and Hoxa11 are within both the same 1MB bin and the same 10kb bin, so they are assigned the same color.

For manuscripts utilizing custom algorithms or software that are central to the research but not yet described in published literature, software must be made available to editors and reviewers. We strongly encourage code deposition in a community repository (e.g. GitHub). See the Nature Portfolio guidelines for submitting code & software for further information.

Data

Policy information about availability of data

All manuscripts must include a data availability statement. This statement should provide the following information, where applicable:

- Accession codes, unique identifiers, or web links for publicly available datasets
- A description of any restrictions on data availability
- For clinical datasets or third party data, please ensure that the statement adheres to our policy

All sequencing data generated here are uploaded to GEO. Accession number of this series is GSE218274 (https://www.ncbi.nlm.nih.gov/geo/query/acc.cgi?acc=GSE218274). St. Jude ProteinPaint RNA sequencing data accessed through their open resource page (https://pecan.stjude.cloud).

Research involving human participants, their data, or biological material

Policy information about studies with human participants or human data. See also policy information about sex, gender (identity/presentation), and sexual orientation and race, ethnicity and racism.

Reporting on sex and gender	N/a
Reporting on race, ethnicity, or other socially relevant groupings	N/a
Population characteristics	N/a
Recruitment	N/a

Ethics oversight | N/a

Note that full information on the approval of the study protocol must also be provided in the manuscript.

Field-specific reporting

Please select the one below that is the best fit for your research. If you are not sure, read the appropriate sections before making your selection.

☒ Life sciences ☐ Behavioural & social sciences ☐ Ecological, evolutionary & environmental sciences

For a reference copy of the document with all sections, see nature.com/documents/nr-reporting-summary-flat.pdf

Life sciences study design

All studies must disclose on these points even when the disclosure is negative.

Sample size

In previously published work we determined that a sample size of between 18 and 23 had generated sufficient power and so we targeted similar or larger sample sizes with our mouse breeding strategies and included both males and females.

For non-mouse experiments, no sample size calculation was performed a priori. We strove to acquire a minimum of three replicates per experiment to provide a large enough sample size to analyze the data using statistical tests. Any result only observed in duplicate is shown as such, without statistical analyses. Exact sample size for all data shown is described within the Methods, the Figures, or the Figure Legends. We performed analyses in a similar manner in regard to sample size and statistical analyses as per our prior studies (Landgon et al., Nature Communications, 2021; Hanna et al., J Pathology, 2022; Hanna et al., Oncogene, 2018; Drummond et al. Cancer Cell, 2018; Hanna et al., Cancer Research, 2017; Hanna et al., Cell Death and Disease, 2016).

Data exclusions

No data were excluded

Replication

All results were observed at least in duplicate and confirmed to be successful

Randomization

All samples were randomly assigned

Blinding

For in vitro and in vivo studies blinding was not possible because investigators performed and analyzed the experiments.

For pathological assessment a veterinary pathologist who did not perform the experiments was blinded to the mouse genotypes during assessment.

Only essential metadata was provided to computational biologists in order to perform analyses

Reporting for specific materials, systems and methods

We require information from authors about some types of materials, experimental systems and methods used in many studies. Here, indicate whether each material, system or method listed is relevant to your study. If you are not sure if a list item applies to your research, read the appropriate section before selecting a response.

Materials & experimental systems

n/a	Involved in the study	
☐	☒	Antibodies
☐	☒	Eukaryotic cell lines
☒	☐	Palaeontology and archaeology
☐	☒	Animals and other organisms
☒	☐	Clinical data
☒	☐	Dual use research of concern
☒	☐	Plants

Methods

n/a	Involved in the study	
☒	☐	ChIP-seq
☐	☒	Flow cytometry
☒	☐	MRI-based neuroimaging

Antibodies

Antibodies used

Immunostaining
Laminin (Frozen) L9393, Sigma 1:250
Pax7 (Frozen) Purified from DSHB hybridoma, 1:250
MyHC (Frozen) MF20, DSHB, undiluted
Desmin (FFPE) RB-9014, ThermoFisher 1:500
MyoD1 (FFPE) 386R-18, Clone EP212, Cell Marque, undiluted
Myogenin (FFPE) M3559, Clone F5D, DAKO 1:200

Ki67 (FFPE) RM-9106, Clone SP6, ThermoFisher, 1:100
CD31 (Fresh Fixed) ab28364, abcam 1:50
MyoD1 (Fresh Fixed) M3512, Clone 5.8A DAKO 1:50
RFP/Tomato (Fresh Fixed), 600-401-379, Rockland 1:100
ERG (FFPE) ab133264, abcam 1:1700
CD31 (FFPE) DIA-310, Clone SZ31, Histobiotec 1:50
CD5 (FFPE) 553017, Clone 53-7.3, BD Biosciences 1:200
OSCAR (FFPE) 465-01 Covance 1:250
IBA1 (FFPE) CP290A, BioCare Medical 1:300
MAC2 (FFPE) ACL-8942P, Clone M3/M8 Accurate 1:1000
CD3 (FFPE) sc-1127, Clone M-20,Santa Cruz 1:500
PAX5 (FFPE) ab109443, Clone EPR3730(2), Abcam 1:1000
IRF4 (FFPE) sc-6059, Santa Cruz 1:4000
KAPPA (FFPE) 1050-01, Southern Biotechnology Associates, 1:2,500
CD117 (FFPE) AF1356, R&D 1:250
Mast Cell Trypase (FFPE) ab151757, Clone EPR9522, Abcam 1:6000
MCPT4 (FFPE) LS-B5958, LIFESPAN BIO 1:500
GLYP3 (FFPE) 760-4442, CELL MARQUE, RTU
CK19 (FFPE) TROMA-III-C, BANK UNIVERSITY OF IOWA, 1:1000
AFP (FFPE) A0008, DAKO, 1:300
Immunobloting
RFP/Tomato, 600-401-379, Rockland 1:1000 5% BSA
GAPDH, Clone 6C5, MAB374, Sigma-Aldrich, 1:10,000 5% milk
T Antigen, Clone Pab 101, sc-147, Santa Cruz, 1:200 5% milk
hTERT, 600-401-252S, Rockland, 1:500 5% BSA
MYCN, Clone D1V2A, 84406, CST, 1:1,000 5% BSA
FOXO1, Clone 29H4, 2880, CST, 1:1,000 5% BSA
MYOD1, Clone 5.8A, M3512, DAKO, 1:5,000 5% milk
MYOG, Clone F5D, M3559, DAKO, 1:250 5% milk
Flow Cytometry
MAC1, Clone M1/70, #563015, BD Biosciences, 1:50
CD45, Clone I3/2.3, #567459, BD Biosciences, 1:50
TER199, Clone TER-119, #747740, BD Biosciences, 1:50
SCA1, Clone D7, #25-5981-82, eBioscience, 1:50
B1 Integrin, Clone Ha2/5, #561796, BD Biosciences, 1:50
CXCR4, Clone 2B11/CXCR4, #551968, BD Biosciences, 1:50
Streptavidin, #25-431882 eBioscience, 1:50
CD31, Clone 8G12, #340430, BD Biosciences, 1:6
CD34, Clone WM59, #555445, BD Biosciences, 1:6
VE-CADHERIN, Clone 16B1, #17-1449-42, Invitrogen, 1:6
CUT&RUN
H3K27ac, 39133, Active Motif, 1:50
IgG, NB810-56910, Novus, 1:100
FOXO1, Clone 29H4, 2880, CST, 1:50

Validation

Immunostaining
Laminin (Frozen) L9393, Sigma 1:250, manufacturer's validation (Enhanced Validation by Independent Antibodies; L99393 showed similar staining pattern as previously Identified laminin antibody SAB4200719)

Pax7 (Frozen) Purified from DSHB hybridoma, 1:250, manufacturer's validation (Validated via epitope mapping: C-term region aa 418-427; Aloisio et al. JCI 124.9 (2014 Sep): 3929-44.)

MyHC (Frozen) MF20, DSHB, undiluted, manufacturer's validation (Validated via epitope mapping: 92 nm from MHC C-terminus; Shimizu et al. J. of Cell Bio 101.3 (1985 Sep): 1115-23.)

Desmin (FFPE) RB-9014, ThermoFisher 1:500, manufacturer's validation (Validation via epitope mapping: Maps near C-terminus of human desmin protein) and in-house by Comparative Pathology Core

MyoD1 (FFPE) 386R-18, Clone EP212, Cell Marque, undiluted, manufacturer's validation (Certified for in vitro diagnostic use. Validation through use in diagnosis of RMS, which strongly overexpresses MyoD1; Sebire, N J, and M Malone. "Myogenin and MyoD1 expression in paediatric rhabdomyosarcomas." Journal of clinical pathology vol. 56,6 (2003): 412-6.) and in-house by Comparative Pathology Core

Myogenin (FFPE) M3559, Clone F5D, DAKO 1:200, manufacturer's validation (Validation via epitope mapping: maps to aa 138-158 and labels human cultured myotubes; W.E. Wright, Molecular and cellular biology 11.8 (1991 Aug): 4104-10.) and in-house by Comparative Pathology Core.

Ki67 (FFPE) RM-9106, Clone SP6, ThermoFisher, 1:100, manufacturer's validation (Certified for in vitro diagnostic use. Validation provided in :Keszthelyi R et al. Immunomorphological Assessment of the New Ki-67 Specific Rabbit Monoclonal SP6 Antibody on Fixed-embedded Tissue Sections. Submitted for publication in Pathology Oncology Research.) and in-house by Comparative Pathology Core

CD31 (Fresh Fixed) ab28364, abcam 1:500, manufacturer's validation (Validated by observing reactivity only in regions as expected in positive control tissue (human tonsil tissue)) and in-house by Comparative Pathology Core

MyoD1 (Fresh Fixed) M3512, Clone 5.8A, DAKO 1:250, manufacturer's validation (Validation through use in diagnosis of RMS, which

strongly overexpresses MyoD1;Cui S, et al. Evaluation of new monoclonal anti-MyoD1 and anti-myogenin antibodies for the diagnosis of rhabdomyosarcoma. Pathol Int. 1999; 49:62-8.)

RFP/Tomato, 600-401-379, Rockland 1:100, manufacturer's validation (Validation via crossed immunoelectrophoresis: "immunoelectrophoresis resulted in a single precipitation arc against anti-Rabbit Serum and purified and partially purified Red Fluorescent Protein (Discosoma). No reaction was observed against Human, Mouse or Rat serum proteins.")

ERG (FFPE) ab133264, abcam 1:1700, manufacturer's validation (Validated by observing specific reactivity in expected regions in fixed human prostate adenocaricoma, colonic carcinoma, and in Western Blots containing lysate from Jurkat, 293T, and MCF7 cells) and in-house by Comparative Pathology Core

CD31 (FFPE) DIA-310, Clone SZ31, Histobiotec 1:50, manufacturer's validation (Validated by observing specific reactivity in expected regions (vessels and capillaries) in fixed murine aorta, endocardium, small intestine, colon, brain, lymph nodes, and bone marrow) and in-house by Comparative Pathology Core

CD5 (FFPE) 553017, Clone 53-7.3, BD Biosciences 1:200, manufacturer's validation (Validation provided in van Ewijk W, van Soest PL, van den Engh GJ. Fluorescence analysis and anatomic distribution of mouse T lymphocyte subsets defined by monoclonal antibodies to the antigens Thy-1, Lyt-1, Lyt-2, and T-200. J Immunol. 1981; 127(6):2594-2604 and Ledbetter JA, Herzenberg LA. Xenogeneic monoclonal antibodies to mouse lymphoid differentiation antigens. Immunol Rev. 1979; 47:63-90.) and in-house by Comparative Pathology Core

OSCAR (FFPE) 465-01 Covance 1:250, manufacturer's validation (Validation by only observing reactivity only in regions as expected in positive control tissue (squamouse cells surrounding sebaceous cells in skin);Gown, AM, Yaziji H, Barry TS, Hwang HC. OSCAR, A novel broad anti-cytokeratin monoclonal antibody optimized for diagnostic immunohistochemistry. US Canadian Acad Pathol Ann Meeting, 2003.) and in-house by Comparative Pathology Core.

IBA1 (FFPE) CP290A, BioCare Medical 1:300, manufacturer's validation (Certified for in vitro diagnostic use. Validation by observing specific reactivity in microglia. Certified for in vitro diagnostic use; Ito D, et al. Microglia-specific localization of a novel calcium binding protein, Iba1. Brain Res Mol Brain Res. 1998 Jun 1;57(1):1-9.) and in-house by Comparative Pathology Core

MAC2 (FFPE) ACL-8942P, Clone: M3/M8, Accurate 1:1000, manufacturer's validation (Validation provided at Ho MK. and Springer TA. 1982. J. Immunol. 128:1221 and Rosenberg I, et al. 1991. J. Biol. Chem. 266:18731.) and in-house by Comparative Pathology Core

CD3 (FFPE) sc-1127, Santa Cruz 1:500, manufacturer's validation(Validation via epitope mapping: maps to C-terminus of CD3-epsilon) and in-house by Comparative Pathology Core

PAX5 (FFPE) ab109443, Clone EPR3730, Abcam 1:1000, manufacturer's validation (Validated by observing reactivity only in regions as expected in positive control tissue (human tonsil tissue and rat spleen)) and in-house by Comparative Pathology Core

IRF4 (FFPE) sc-6059, Santa Cruz 1:4000, manufacturer's validation(Validated by epitope mapping: maps to C-terminus of IRF-4) and in-house by Comparative Pathology Core

KAPPA (FFPE) 1050-01, Southern Biotechnology Associates, 1:2,500, manufacturer's validation (Validation provided in Hemminki A, Niemi S, Hoffrén A, Hakalahti L, Söderlund H, Takkinen K. Specificity improvement of a recombinant anti-testosterone Fab fragment by CDRIII mutagenesis and phage display selection. Protein Eng. 1998;11:311-9.) and in-house by Comparative Pathology Core.

CD117 (FFPE) AF1356, R&D 1:250, manufacturer's validation (Validated by observing specific reactivity in expected regions in murine embryos and in Western Blots containing lysate from P815 mouse mastocytoma cells and MC/9-2 mouse mast cells) and in-house by Comparative Pathology Core

Mast Cell Trypase (FFPE) ab151757, Clone EPR9522, Abcam 1:6000, manufacturer's validation (Validated by observing specific reactivity in expected regions in fixed human tissue and in Western Blots containing lysate from human skin, colon cancer, and tonsil cells) and in-house by Comparative Pathology Core

MCPT4 (FFPE) LS-B5958, LIFESPAN BIO 1:500, manufacturer's validation (Validated via epitope mapping: maps to aa 114-126) and in-house by Comparative Pathology Core.

GLYP3 (FFPE) 760-4442, CELL MARQUE, RTU, manufacturer's validation (Certified for in vitro diagnostic use; see Kandil DH, et al. Glypican-3 a novel diagnostic marker for hepatocellular carcinoma and more. Adv Anat Pathol. 2009; 16:125-9.) and in-house by Comparative Pathology Core.

CK19 (FFPE) TROMA-III-C, BANK UNIVERSITY OF IOWA, 1:1000, manufacturer's validation (Validation provided here: Brulet et al., Monoclonal antibodies against trophectoderm-specific markers during mouse blastocyst formation. Proceedings of the National Academy of Sciences of the United States of America 77.7 (1980 Jul): 4113-7.) and in-house by Comparative Pathology Core

AFP (FFPE) A0008, DAKO, 1:300, manufacturer's validation (Validation performed via crossed immunoelectrophoresis: "only the alpha-1-fetoprotein precipitation arch appears when using 12.5 uL A0008 antibody per cm^2 gel area against 2 uL of a human cord serum fraction. No precipitation arch is seen when the antibody is tested against 2 µL human plasma. Staining: Coomassie Brilliant Blue.") and in-house by Comparative Pathology Core

Immunoblotting
RFP/Tomato, 600-401-379, Rockland 1:1000 5% BSA, manufacturer's validation (Validation via crossed immunoelectrophoresis: "immunoelectrophoresis resulted in a single precipitin arc against anti-Rabbit Serum and purified and partially purified Red Fluorescent Protein (Discosoma). No reaction was observed against Human, Mouse or Rat serum proteins.")

GAPDH, Clone 6C5, MAB374, Sigma-Aldrich, 1:10,000 5% milk, manufacturer's validation (Validated by observing specific reactivity in

expected regions in fixed Hela and NIH3T3 cells and in Western Blots containing lysate from A431, C2C12, HEK293, Hela, GepG2, human placenta, HUVEC, and L6 cells.)

T Antigen, Clone Pab 101, sc-147, Santa Cruz, 1:200 5% milk, manufacturer's validation (Validation via epitope mapping: maps to the C-terminus of Large T Antigen; see Antigenic binding sites of monoclonal antibodies specific for simian virus 40 large T antigen, Gurney, EG., et al. 1986. J Virol. 57: 1168-72. PMID: 2419584)

hTERT, 600-401-252S, Rockland, 1:500 5% BSA, manufacturer's validation (Validated by observing specific reactivity in expected regions in fixed fibroblasts exposed to H202 cells and in Western Blots containing lysate from HeLa cell transduced with an hTERT expressing vector)

MYCN, Clone D1V2A, 84406, CST, 1:1,000 5% BSA, manufacturer's validation (Validated by observing specific reactivity in expected regions in fixed IMR-32 cells and in Western Blots containing lysate from IMR-32 cells)

FOXO1, Clone 29H4, 2880, CST, 1:1,000 5% BSA, manufacturer's validation (Validation by observing specific reactivity in expected regions in Western Blots containing lysate from wild-type 293T cells. No band detected in FOXO-KO control 293Ts).

MYOD1, Clone 5.8A, M3512, DAKO, 1:5,000 5% milk, manufacturer's validation (Validation through use in diagnosis of RMS, which strongly overexpresses MyoD1;Cui S, et al. Evaluation of new monoclonal anti-MyoD1 and anti-myogenin antibodies for the diagnosis of rhabdomyosarcoma. Pathol Int. 1999; 49:62-8.)

MYOG, Clone F5D, M3559, DAKO, 1:250 5% milk, manufacturer's validation (Validation via epitope mapping: maps to aa 138-158 and labels human cultured myotubes; W.E. Wright, Molecular and cellular biology 11.8 (1991 Aug): 4104-10.)

Flow Cytometry
MAC1, Clone M1/70, #563015, BD Biosciences, 1:50, manufacturer's validation (Validation provided in Ault KA, Springer TA. Cross-reaction of a rat-anti-mouse phagocyte-specific monoclonal antibody (anti-Mac-1) with human monocytes and natural killer cells. J Immunol. 1981; 126(1):359-364.)

CD45, Clone I3/2.3, #567459, BD Biosciences, 1:50, manufacturer's validation (Validation provided in Lefrancois L, Goodman T. Developmental sequence of T200 antigen modifications in murine T cells.. J Immunol. 1987; 139(11):3718-24.; Johnson P, Greenbaum L, Bottomly K, Trowbridge IS. Identification of the alternatively spliced exons of murine CD45 (T200) required for reactivity with B220 and other T200-restricted antibodies. J Exp Med. 1989; 169(3):1179-1184.;Trowbridge IS. Interspecies spleen-myeloma hybrid producing monoclonal antibodies against mouse lymphocyte surface glycoprotein, T200.. J Exp Med. 1978; 148(1):313-23.)

TER199, Clone TER-119, #747740, BD Biosciences, 1:50, manufacturer's validation (Validation provided in Kina T, Ikuta K, Takayama E, et al. The monoclonal antibody TER-119 recognizes a molecule associated with glycophorin A and specifically marks the late stages of murine erythroid lineage. Br J Haematol. 2000; 109(2):280-287.)

SCA1, Clone D7, #25-5981-82, eBioscience, 1:50, manufacturer's validation (Validation in C57BL/6 splenocytes, compared to Rat IgG2a K Isotype Control)

B1 Integrin, Clone Ha2/5, #561796, BD Biosciences, 1:50, manufacturer's validation (Validation provided in Mendrick DL, Kelly DM. Temporal expression of VLA-2 and modulation of its ligand specificity by rat glomerular epithelial cells in vitro. Lab Invest. 1993; 69(6):690-702.)

CXCR4, Clone 2B11/CXCR4, #551968, BD Biosciences, 1:50, manufacturer's validation (Validation provided in Forster R, Kremmer E, Schubel A, et al. Intracellular and surface expression of the HIV-1 coreceptor CXCR4/fusin on various leukocyte subsets: rapid internalization and recycling upon activation. J Immunol. 1998; 160(3):1522-1531.)

Streptavidin, #25-431882 eBioscience, 1:50, manufacturer's validation (Validation through staining C57BL/6 splenocytes with Anti-Mouse CD3e Biotin, followed by 0.06 µg of Streptavidin PE-Cyanine7 and detecting specific signal in live cells)

CD31, Clone WM-59 , #340430, BD Biosciences, 1:6, manufacturer's validation (Validated via epitope mapping: maps to an epitope proximal to extracellular domain 2 of CD31)

CD34, Clone 8G12, #555445, BD Biosciences, 1:6, manufacturer's validation (Validated via epitope mapping: maps to 3 epitopes on CD34, all distinct from the ones bound by clone My10)

VE-CADHERIN, Clone 16B1, #17-1449-42, Invitrogen, 1:6, manufacturer's validation (Validated by comparing staining in HUVECs to Mouse IgG1 kappa Isotype Control)

CUT&RUN
H3K27ac, 39133, Active Motif, 1:50, manufacturer's validation ("This antibody has been validated for CUT&Tag using Active Motif's CUT&Tag-IT™ Assay Kit, Catalog No. 53160."; Also validated for use in the modENCODE project)

IgG, NB810-56910, Novus, 1:100, manufacturer's validation (Validated by immunoelectrophoresis: "By immunoelectrophoresis the IgG was shown to 1) react with antiserum specific for rabbit IgG, 2) not react with antiserum specific for IgA or IgM, and 3) produce a single precipitin arc with antiserum against rabbit serum identical to that produced with anti-IgG antisera.")

FOXO1, Clone 29H4, 2880, CST, 1:50, manufacturer's validation (Validation by observing specific reactivity in expected regions in Western Blots containing lysate from wild-type 293T cells. No band detected in FOXO-KO control 293Ts).

Eukaryotic cell lines

Policy information about cell lines and Sex and Gender in Research

Cell line source(s)	293T (Martine Roussel, SJCRH) cells were maintained in DMEM (#SH32043, HyClone) supplemented with 10% fetal bovine serum (FBS, HyClone) and 1% antibiotic/antimycotic . HUVECs (LifeLine Cell Technologies, FC-0044) were maintained in VascuLife VEGF Endothelial Medium Complete Kit (LifeLine Cell Technologies, LL-0003). Primary HUVECs were immortalized by retroviral transduction with large T (genomic) antigen (#1778, Addgene, Bob Weinberg) and telomerase reverse transcriptase (pBABE-hygro-hTERT) (#1773, Addgene). BJFF.6 Human iPSCs were maintained in mTeSR Plus (Stemcell Technologies, 100-0276) on growth factor reduced Matrigel basement membrane matrix (Corning, 354230). All cell lines were maintained in a humidified incubator at constant 37 °C and 5% CO_2.
Authentication	Cell lines authenticated by short tandem repeat profiling and compared to known profiles. Pluripotency of BJFF.6 iPSCs was determined by teratoma formation.
Mycoplasma contamination	Cell lines routinely monitored for mycoplasma contamination (Universal Mycoplasma Detection Kit, ATCC, #30-1012K) and were all mycoplasma-negative.
Commonly misidentified lines (See ICLAC register)	No misidentified lines used.

Animals and other research organisms

Policy information about studies involving animals; ARRIVE guidelines recommended for reporting animal research, and Sex and Gender in Research

Laboratory animals	All mouse strains are reported: aP2-Cre, R26-tdTomato (#7914, The Jackson Laboratory (JAX), Pax3Pax3-Foxo1, Cdkn2aFlox (Nabeel Bardeesy), Fabp4-Cre (#5069, The Jackson Laboratory), Tek-Cre35, Myf6-Cre. For Kaplan-Meier survival analysis, animals were observed from birth and sacrificed at humane endpoints, such as showing signs of obvious tumor burden or other distress. Full necropsies were performed. All mice had consistent access to food and water and were housed at ambient temperature (20-25 C) and humidity (40-60%) with 12-h light/12-h dark cycles.<br><br>Human cell line xenografts implanted into SCID/Beige (#250, Charles River Laboratories) mice. Mice were >6 weeks of age at the time of engraftment.
Wild animals	Study did not use wild animals
Reporting on sex	Our findings apply to both sexes, as both male and female mice were used in collection of all murine data. Sex as a biological variable was not explicitly considered in study design, although we planned to include animals of both sexes in all experiments. We did not include or exclude results based on the sex of animals used. Vaginal-bearing mice were assigned female and penis-bearing mice were assigned male. Data disaggregated for sex (where collected) has been provided in the source data. Information on sex of the animals in Kaplan-Meier curves shown in Supp Fig 1a (ACP tumor-free survival), Supp Fig 1q (FCP tumor-free survival), and Supp Fig 3e (TC tumor-free survival) is incomplete due to reporting errors.<br>Overall numbers in tumor-free survival curves are as follows:<br><br>TCP Females: 11<br>TCP Males: 12<br>MCP Males: 19<br>MCP Females: 21<br>ACP Males: 7<br>ACP Females: 11<br>ACP Unreported: 5<br>FCP Males: 2<br>FCP Females: 11<br>FCP Unreported: 3<br>TC Males: 2<br>TC Females: 0<br>TC Unreported: 8<br><br>There was no statistically significant differences in the tumor onset between TCP males and females (Log-rank test P = 0.2491), TCP males and MCP males (Log-rank test P = 0.8489), TCP females and MCP females (Log-rank test P = 0.6027). MCP males displayed a ~15% faster tumor onset compared to MCP females (Log-rank test P = 0.0227). We have not performed sex-based analyses on the other genotypes due to unreported sexes.
Field-collected samples	No field collected samples were used
Ethics oversight	The work reported here involving vertebrate animals was approved by the St. Jude Children's Research Hospital Institutional Animal Care and Use Committee

Note that full information on the approval of the study protocol must also be provided in the manuscript.

Plants

Seed stocks	N/a
Novel plant genotypes	N/a
Authentication	N/a

Flow Cytometry

Plots

Confirm that:

☐ The axis labels state the marker and fluorochrome used (e.g. CD4-FITC).

☐ The axis scales are clearly visible. Include numbers along axes only for bottom left plot of group (a 'group' is an analysis of identical markers).

☒ All plots are contour plots with outliers or pseudocolor plots.

☒ A numerical value for number of cells or percentage (with statistics) is provided.

Methodology

Sample preparation	Tumors were dissected, manually dissociated, and digested for 1 hour at 37°C in 2 U/mL Collagenase B (#11088831001, Roche)/Dispase II (#04942078001, Roche), 50 mM HEPES/KOH pH 7.4; 150 mM NaCl. Following the addition of 2X volume 10% fetal bovine serum (FBS) (#SH30910.03, GE Hyclone) in phosphate buffered saline (PBS) to inactive digestion enzymes, samples were sequentially filtered through 70 µm (#22363548, Fisher) and 40 µm (#22363547, Fisher) filters to yield single cell suspensions. Single cells were blocked in 5% FBS in PBS and then stained for 30 minutes on ice.<br><br>iPSCs were dissociated using accutase and filtered with 40 µm filters to yield single cell suspensions. Cells were blocked with 5% FBS in PBS and then stained for 30 minutes on ice.
Instrument	Flow cytometry analysis for endothelial differentiation markers shown in Supplementary Figure 9f was performed on a Cytek Aurora spectral analyzer and was analyzed with SpectroFlo. All other flow cytometry analyses were collected using a BD Biosciences Symphony analyzer and analyzed with FlowJo. A BD Biosciences Aria cell sorter was used for cell sorting
Software	FlowJo v10.8.1 and SpectroFlo 3.0.3
Cell population abundance	When purifying the tdTomato positive cells, that population was typically less than half of live single cells.<br><br>The muscle stem cell population (MAC1, CD45, TER119, and SCA1 negative/ β1-Integrin and CXCR4 positive) was less than 5% of total live, single cells.
Gating strategy	tdTom positive and negative: DAPI+ live cells, FSC-A/SSC-W to define single cells, tdTomato positive gate separating tdTomato positive and negative.<br><br>Muscle stem cell sort: DAPI+ live cells, FSC-A/SSC-W to define single cells, lineage (MAC1, CD45, TER119)/SCA1 negative, β1-Integrin/CXCR4 positive

☐ Tick this box to confirm that a figure exemplifying the gating strategy is provided in the Supplementary Information.

This checklist template is licensed under a Creative Commons Attribution 4.0 International License, which permits use, sharing, adaptation, distribution and reproduction in any medium or format, as long as you give appropriate credit to the original author(s) and the source, provide a link to the Creative Commons license, and indicate if changes were made. The images or other third party material in this article are included in the article's Creative Commons license, unless indicated otherwise in a credit line to the material. If material is not included in the article's Creative Commons license and your intended use is not permitted by statutory regulation or exceeds the permitted use, you will need to obtain permission directly from the copyright holder. To view a copy of this license, visit http://creativecommons.org/licenses/by/4.0/
