# Supplementary material for: PAX3-FOXO1 dictates myogenic reprogramming and rhabdomyosarcoma identity in endothelial progenitors
Source: Nat Commun. 2023 Nov 15;14:7291. doi: 10.1038/s41467-023-43044-1 (PMC10651858; doi:10.1038/s41467-023-43044-1)
Supplement: Supplementary file 32 — Description of Additional Supplementary Files [file 41467_2023_43044_MOESM32_ESM.pdf]

Title: Supplementary Data 1:
Description: List of antibodies used for staining

Title: Supplementary Data 2:
Description: Clusters from *aP2-Cre;R26-tdTom* and *Fabp4-Cre;R26tdTom* scRNAseq.

Title: Supplementary Data 3:
Description: Clusters from stringent Tomato positive *aP2-Cre;R26-tdTom* scRNAseq.

Title: Supplementary Data 4:
Description: *Tek-Cre;R26-tdTom* Tom+ and Tom- RNAseq.

Title: Supplementary Data 5:
Description: *Tek-Cre;R26-tdTom* Tom+ and Tom- GSEA.

Title: Supplementary Data 6:
Description: *aP2-Cre;R26-tdTom* and *Tek-Cre;R26-tdTom* Tom+ RNAseq.

Title: Supplementary Data 7:
Description: *aP2-Cre;R26-tdTom* Tom+ enriched GO terms.

Title: Supplementary Data 8:
Description: *Tek-Cre;R26-tdTom* Tom+ enriched GO terms.

Title: Supplementary Data 9:
Description: *aP2-Cre;R26-tdTom* and *Tek-Cre;R26-tdTom* Tom+ shared GO terms.

Title: Supplementary Data 10:
Description: Clusters *aP2-Cre;R26-tdTom* and *Tek-Cre;R26-tdTom* scRNAseq.

Title: Supplementary Data 11:
Description: RNAseq from bulk TCP and MCP tumors.

Title: Supplementary Data 12:
Description: TCP compared to ARMS PCGP GSEA.

Title: Supplementary Data 13:
Description: MCP compared to ARMS PCGP GSEA.

Title: Supplementary Data 14:
Description: RMS genes

Title: Supplementary Data 15:
Description: Clusters from TCP tumors scRNAseq.

Title: Supplementary Data 16:
Description: Clusters from MCP tumors scRNAseq.

Title: Supplementary Data 17:
Description: Clusters from combined TCP, MCP, total muscle, MuSCs, Primary MBs and *Tek-Cre;R26-tdTom* scRNAseq.

Title: Supplementary Data 18:
Description: CellMarker Augmented 2021 terms from cluster 8.

Title: Supplementary Data 19:
Description: Clusters from reclustered tumor population from combined TCP, MCP, muscle and *Tek-Cre;R26-tdTom* scRNAseq.

Title: Supplementary Data 20:
Description: PanglaoDB Augmented 2021 terms from cluster 0.

Title: Supplementary Data 21:
Description: PanglaoDB Augmented 2021 terms from cluster 6.

Title: Supplementary Data 22:
Description: Mouse genes associated with RMS cell states.

Title: Supplementary Data 23:
Description: H3K27ac peaks and associated genes.

Title: Supplementary Data 24:
Description: Differential H3K27ac peak coverage in MCP vs TCP.

Title: Supplementary Data 25:
Description: GSEA on unique H3K27ac peaks in TCP vs MCP tumors.

Title: Supplementary Data 26:
H3K27ac super enhancer peaks and associated genes.

Title: Supplementary Data 27:
DESeq2 H3K27ac super enhancer associated genes in MCP vs TCP.

Title: Supplementary Data 28:
GSEA on unique super-enhancer associated genes in TCP vs MCP tumors.

Title: Supplementary Data 29:
Endothelial cell transcription factors.
